# Systematic Literature Review of the Effect of Layered Double Hydroxides on the Mechanical Properties of Rubber

**DOI:** 10.3390/polym13213716

**Published:** 2021-10-28

**Authors:** Louise van Tonder, Frederick Johannes Willem Jacobus Labuschagné

**Affiliations:** Department of Chemical Engineering, University of Pretoria, Lynnwood Rd, Hatfield, Pretoria 0002, South Africa; johan.labuschagne@up.ac.za

**Keywords:** layered double hydroxide, layered nanoclay, elastomer, mechanical properties, composites

## Abstract

Layered double hydroxides (LDHs) have attracted interest as reinforcing fillers in elastomers due to their ease of synthesis and customisability. A systematic review was performed on the effect of LDHs on the mechanical properties of elastomers using the Scopus database. Of the 61 articles relevant to the search criteria, the majority were published on polyurethane (PU) and nitrile butadiene rubber (NBR). Mg-Al LDH was used in most of the studies and Zn-Al LDH was used second most common. LDH can act as a reinforcing filler, typically increasing tensile strength even at low concentrations, so it could be used as an alternative to traditional reinforcing fillers for elastomers. LDH can also be made a functional filler by selecting the right metals and interlayer anions. It was found that Mg-Al LDH and Zn-Al LDH can both participate in crosslinking reactions and can replace MgO and ZnO, respectively. Less Zn ions are required for crosslinking when LDH is used than when ZnO is used, making LDH more environmentally friendly. Organic modification is usually required to improve compatibility with the elastomer matrix, especially in non-polar elastomers. It enables exfoliation of the LDH and intercalation of polymer chains into the LDH interlayer to occur. Organic modifiers can also be used to functionalise the LDH. Stearic acid used in crosslinking systems can be replaced by stearate anions from stearate-modified LDH.

## 1. Introduction

Elastomers are polymers that can typically undergo chemical reactions after being heated to form bonds or crosslinks between the polymer chains, forming a flexible polymer network [1]. This network structure allows them to undergo large elastic deformations before breaking [2]. Elastomers have a wide range of applications, such as car and truck tires, conveyor belts, seals, hoses and many others. For use in many of these applications, elastomers also need to be reinforced to enhance their mechanical properties. Commonly used reinforcing fillers include carbon black and silica combined with a silane coupling agent [3]. The crosslinks, filler reinforcing effect and filler–polymer interaction all influence the mechanical properties of the composite. In recent years other reinforcing fillers, such as montmorillonite (MMT) and layered double hydroxides (LDHs) have been attracting attention.

Both LDH and MMT are layered clays. MMT is a cationic silicate clay that consists of thin layers of tetrahedral silica sheets sandwiching an octahedral alumina sheet [4]. Layered silicates such as montmorillonite have long been used as fillers in polymers [5,6] and in elastomers for some time [7,8,9]. LDH has also gained more attention as a filler for elastomers. While LDH has been used in thermoplastic polymers as additives for several years [10,11,12], their use in elastomers has only been studied more recently. LDH has the advantage over MMT of being very customisable [13]. Both can be organically modified but the interlayer anions, metals and metal ratios of LDH can all be changed [14].

Basu et al. [15] reviewed the work done with LDHs in elastomers up to 2014 and discussed what had been done so far. They discussed the full spectrum of work on LDH in elastomers. LDH can be used to enhance the mechanical, thermal and optical properties as well as improve flame retardancy. Organic modification tended to further improve the properties by improving polymer–filler compatibility. The elastomers that were looked at included carboxylated nitrile rubber (XNBR), etyhylene propylene diene monomer (EPDM), chloroprene rubber (CR), silicone rubber (SR), ethylene vinyl acetate (EVA), poly(1-butene), nitrile butadiene rubber (NBR), polyurethane (PU)/NBR blends and solution styrene butadiene rubber (SSBR). Most of the work focused on Mg-Al LDH although Zn-Al LDH garnered a lot of interest due to its ability to act as a replacement of ZnO in crosslinking systems.

More research has been conducted on LDH in elastomers since the previous review was published. This systematic literature review focuses on how LDH various types of LDH have been used to affect the mechanical properties of elastomers. EVA was excluded from this search as it does not always behave as an elastomer. Other effects of LDH on elastomers, such as flame retardancy, were also not included. Only the Scopus database was used to perform the search.

## 2. Search Criteria and Review Methodology

A systematic approach was taken to search for literature available on the effect of LDHs on the mechanical properties of elastomers. The following terms were used to perform the Scopus database search:

TITLE-ABS-KEY((“LDH” OR “layered double hydroxide” OR “hydrotalcite” OR “hydrocalumite” OR “double layered hydroxide”) AND (“elastomer” OR “rubber” OR “vulcanizate” OR “vulcanisate” OR “natural rubber” OR “NR” OR “epoxidized natural rubber” OR “epoxidised natural rubber” OR “ENR” OR “styrene-butadiene rubber” OR “styrene-butadiene copolymer” OR “SBR” OR “solution styrene-butadiene rubber” OR “SSBR” OR “butadiene rubber” OR “polybutadiene” OR “PBR” OR “ethylene-propylene monomer” OR “EPM” OR “ethylene-propylene-diene monomer” OR “EPDM” OR “polyisoprene” OR “isoprene rubber” OR “acrylonitrile-butadiene copolymer” OR “nitrile rubber” OR “acrylonitrile butadiene rubber” OR “nitrile butadiene rubber” OR “NBR” OR “isobutylene-isoprene copolymer” OR “butyl rubber” OR “chloroprene” OR “polychloroprene” OR “neoprene” OR “polysulfide rubber” OR “thiokol” OR “polydimethyl siloxane” OR “silicone rubber” OR “fluoroelastomer” OR “perfluoroelastomer” OR “polyacrylate elastomer” OR “styrene-isoprene-styrene” OR “styrene-butadiene-styrene block copolymer” OR “SBS” OR “epi-chlorodin rubber” OR “polyacrylic rubber” OR “fluorosilicone rubber” OR “polyether block amide” OR “chlorosulfonated polyethylene” OR “CSM” OR “thermoplastic poly-urethane” OR “polyurethane” OR “TPU” OR “PU” OR “ethylene propylene rubber” OR “thermoplastic polyamide” OR “TPA” OR “carboxylated nitrile rubber” OR “carboxylated nitrile butadiene rubber” OR “XNBR” OR “styrene-ethylene-butylene-styrene” OR “SEBS” OR “hydrogenated nitrile butadiene rubber” OR “HNBR”) AND NOT (“lactate dehydrogenase”)).

LDH is often used to abbreviate “lactate dehydrogenase”, so many articles that contain the abbreviation LDH are not relevant to layered double hydroxides. For this reason the phrase “lactate dehydrogenase” was explicitly excluded in the search criteria to reduce the number of irrelevant articles. Adding the exclusion reduced the number of articles from 538 results to 330 results.

**Figure 1:**
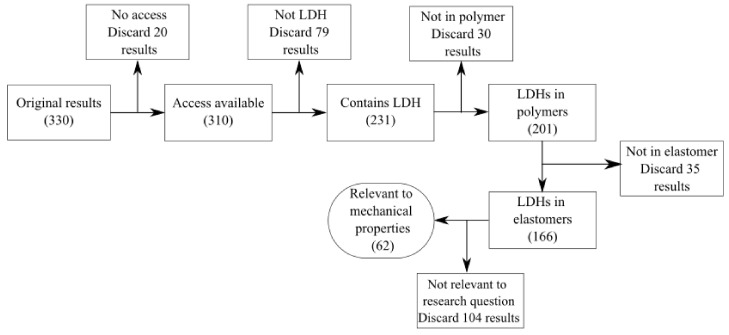
Flowchart of how the literature was refined.

The 330 articles in the Scopus database that matched the search criteria were not, however, all relevant to the research questions. The results were therefore further refined as shown in Figure 1. Due to LDH being used as an abbreviation for terms unrelated to layered double hydroxides in elastomers, several articles that matched the chosen keywords had to be discarded. In some cases LDH was not used in a polymer and these articles were also ignored. After removing results that were unrelated to LDH in elastomers, the final selection comprised only articles related to the mechanical and rheological properties of the elastomers. Articles in which LDH was used as a catalyst, flame retardant, drug delivery system, etc. were removed because, while both elastomers and LDH were present in the articles, the applications were not directly relevant to the research question.

**Figure 2:**
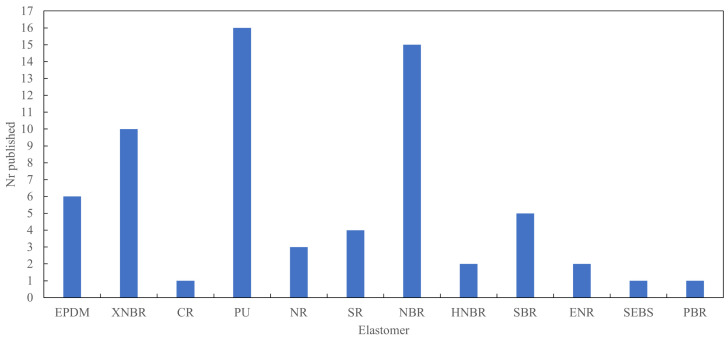
Number of articles published per elastomer type.

The remaining 62 articles were relevant to the question of how LDHs can be used to affect the mechanical properties of elastomers, and these are discussed in detail. One of these results was the previous review article [15] and is not discussed in detail. The literature review therefore consists of the remaining 61 articles. As shown in Figure 2, most of the work was performed on PU, NBR and XNBR. In the case of NBR, four articles that were counted under PU were also counted under NBR due to these two rubbers being used in a 50:50 wt % blend.

**Figure 3:**
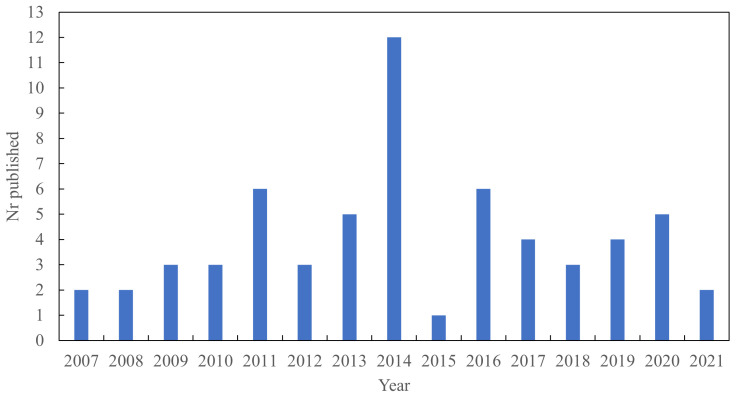
Number of articles published per year.

It was found that the first articles about LDH as a filler in elastomers were published in 2007. Interest in LDH as a reinforcing and functional filler then increased over time, as shown in Figure 3. More recently, there has been a reduction in publications on this topic but several papers are still being published each year in this field.

**Figure 4:**
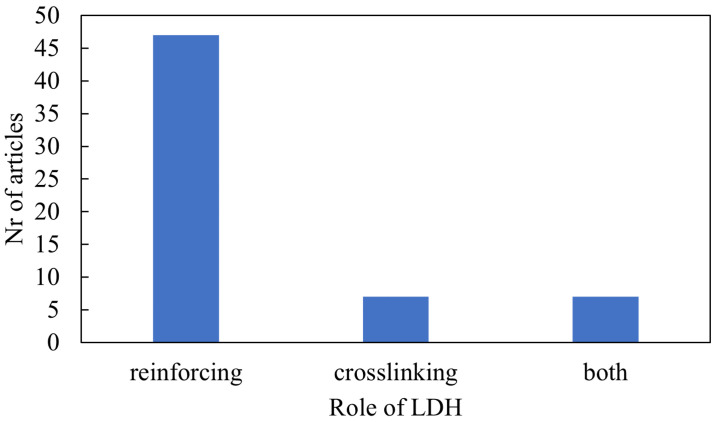
Role of LDH in the elastomers.

**Figure 5:**
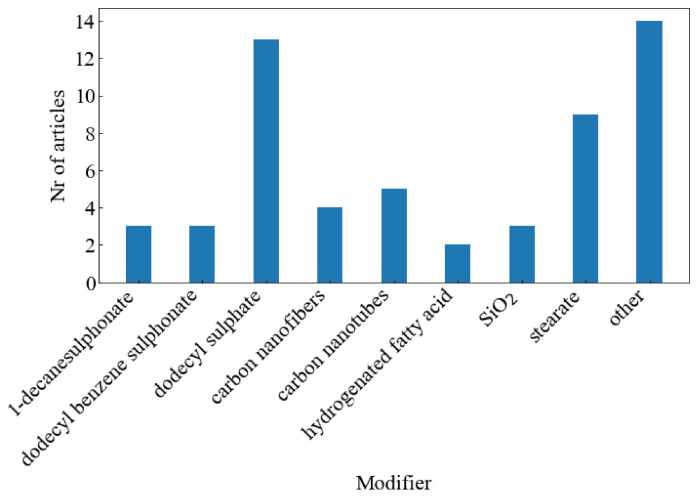
Number of articles in which each modifier was used.

Initial studies focused on the effect of organically modified LDHs on the properties of various elastomers. The organic modifiers were mainly used to increase the LDH interlayer spacing and compatibilise the LDH with the organic polymer matrix. Later on, functional organic modifiers, such as stearic acid, were used so that the LDH could act as both a reinforcing filler and participate in the crosslinking process [16]. Figure 4 shows that LDH usually plays a role as a reinforcing filler, although it can also be used as part of the crosslinking process. The dodecyl sulphate (DS) anion was most often used, as shown in Figure 5, with the stearate (St) anion being used almost as often. The “other” category consists of modifiers that were only used in one study. More recently, other fillers such as carbon nanotubes have been grafted onto LDH.

**Figure 6:**
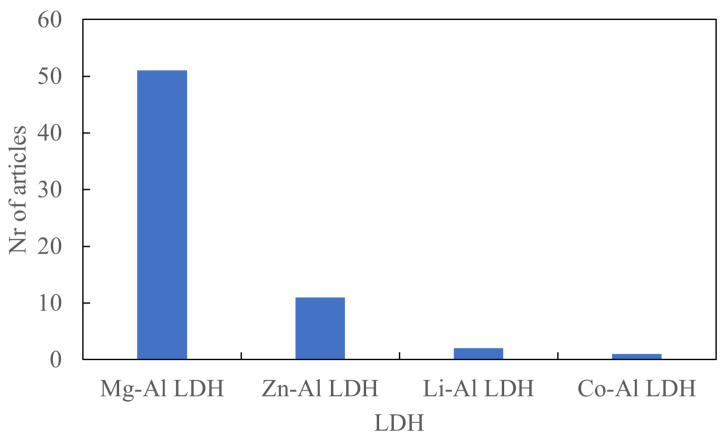
Number of articles in which each type of LDH was used.

Mg-Al LDH is used most often, as shown in Figure 6. The divalent metal, Mg, is sometimes replaced by other metals, since LDHs such as Mg-Al LDH and Zn-Al LDH can be functional in some elastomers [13,16,17].

## 3. Layered Double Hydroxides

As described by Forano et al. [14], an LDH is a layered ionic clay. It has a flat, two-dimensional, brucite-like layered structure, with positively charged metal hydroxide sheets containing anions in the interlayer. The general formula for a layered double hydroxide is [M${}_{1-x}^{II}$M${}_{x}^{III}$(OH)${}_{2}$][X${}_{x/q}^{q-}\cdot$nH${}_{2}$O]. Many divalent and trivalent metals can be used to synthesise LDHs, such as Ca, Al, Mg, Fe, Co, Zn and Cu. Li, despite being a monovalent cation, has also been used to form Li-Al LDH [18]. The most common form of LDH is hydrotalcite, a Mg-Al LDH with the formula Mg${}_{0.75}$Al${}_{0.25}$(OH)${}_{2}$(CO${}_{3}$)${}_{0.5}\cdot$0.5H${}_{2}$O. Ternary combinations of metals can also be used when making synthetic LDHs. Theoretically, any anion can be intercalated into the interlayer of the LDH and even monovalent and tetravalent metals can be used to make LDHs, showing how versatile LDHs can be.

LDH platelets are normally stacked hexagonally or rhombohedrally [19]. The shape and size of LDH nano-particles are affected by the method used to synthesise them. Coprecipitation usually results in particles with a high degree of crystallinity but a wide range of sizes, because nucleation and growth occur simultaneously [14]. Small LDH platelets (between 40 nm and 300 nm) have been synthesised using coprecipitation [20]. Urea hydrolysis can be used to better control particle size, resulting in large hexagonal platelets with a narrow size distribution [21]. LDH platelets tend to stack on top of each other and form agglomerates, which is undesirable in applications such as polymer–LDH nanocomposites [22]. The LDH layers can be exfoliated, creating single layer nano-sheets and increasing the aspect ratio and surface area [23,24]. Exfoliation reduces agglomeration and improves distribution when LDH is incorporated in polymers.

LDHs have found uses in many different fields. They can be used as flame retardants and heat stabilisers [25,26,27], UV stabilisers [28], catalysts [29] and controlled release of drugs and biomolecules [30]. One of the main commercial applications of LDH is as a heat stabiliser and flame retardant in poly(vinylchloride) (PVC) because of its ability to neutralise the acid generated in PVC during heating, reduce acid fumes and sequestrate Cl${}^{-}$ anions [31].

### 3.1. Synthesis of LDH

There are several methods that can be used to synthesise LDH, such as coprecipitation, urea hydrolysis, induced hydrolysis, mechanochemical synthesis and green synthesis [14,32].

Coprecipitation is used most often and involves slowly adding a mixed aqueous solution of the divalent and trivalent metal salts to a solution containing the desired anion [18]. The pH can be kept constant using an alkaline solution. Another approach is to use a variable pH, although the constant pH method produces LDH with better properties [33]. After coprecipitation, the LDH is usually aged to improve the crystallinity.

Urea hydrolysis can be used as an alternative method to coprecipitation to produce large, highly crystalline LDH particles [34]. In this method, the precipitation of the LDH occurs in the presence of urea and heat. The temperature at which synthesis takes place influences how crystalline the LDH is [35].

When the induced hydrolysis method is used, metal oxides or metal hydroxides are added drop-wise to acidic solutions of trivalent metal salts [36]. The oxides are dissolved and LDH precipitates.

As described by Qu et al. [37], mechanochemical synthesis is a more recently developed method that can be performed in three ways, namely single-step grinding; a mechano–hydrothermal process; and two-step grinding, a combination of dry and wet grinding. Metal oxides or hydroxides are ground together using a ball mill. These methods have the advantage over other synthesis methods of being simple and not requiring waste solution treatment. LDH has been synthesised quickly and easily in a one-pot mechanochemical method and has been scaled up successfully [38].

Hydrothermal dissolution–precipitation is a green synthesis method that can be used to produce high yield, high purity LDH [39]. In this process, a metal oxide/hydroxide mixed solution is fed to an autoclave reactor and is allowed to react at a relatively high temperature. The effluent can also be recycled, reducing the expense of waste solution treatment and making scaling-up of this method viable.

### 3.2. LDHs as Filler

Layered nanoclays such as LDH, MMT and other silicate clays are used as fillers in polymers because they have high aspect ratios and surface areas, and can be organically modified. The high aspect ratios and surface areas make the layered clays useful as reinforcing fillers in polymers at lower loadings than reinforcing fillers such as carbon black [40]. The larger surface area increases the interfacial area where polymer–filler interaction takes place, improving interfacial interaction.

While it is harder to exfoliate LDHs, due to the strong interactions between the sheets, than nanoclays such as MMT [41], LDHs have the advantage of being much more flexible as fillers and functional additives than other layered clays. LDHs can be modified easily and can have many different compositions. The type of metals as well as their ratios can be modified, and many anions can be intercalated into the LDH interlayer. This means that the LDH can be customised to match the needs of the application. The metals can, for example, be selected in such a way that they participate in vulcanisation. This was successfully performed by Das et al. [16] where they used a Zn-Al LDH in the place of ZnO to vulcanise various rubbers.

### 3.3. Organic Modification of LDH

LDHs are inorganic and incompatible with non-polar rubbers such as SBR and EPDM. Polar rubbers such as XNBR have groups that can have strong interactions with the hydroxide sheets of the LDHs, making them more compatible. However, even for polar rubbers, without organic modification the interlayer spacing is too narrow to properly exfoliate the clay and intercalate polymer chains or segments [15]. This means the LDH is not an effective reinforcing filler. Organic modification can increase the interlayer spacing of the LDH, making it possible to exfoliate the sheets and intercalate polymer chains into the LDH. This enhances the reinforcing effect of the LDH. The organic modifications also compatibilise the LDH with the organophilic, non-polar rubbers.

Anionic surfactants with long hydrophobic tails can be used to modify the LDH to make it compatible with rubber. Examples of surfactants used for this purpose include dodecyl sulphate, dodecylbenzenesulphonate, laurate and bis(2-ethylhexyl) hydrogen phosphate [42]. Stearate anions can also be intercalated.

Since organic modification is typically required if an LDH is to be used in rubber as a reinforcing filler, it may be useful to select the organic modifier in such a way that it is functional in the rubber. It could, for example, act as an activator or sulphur donor for vulcanisation.

The interlayer anions of LDH can be modified with relative ease using common techniques such as anion exchange and reconstruction. In the anion exchange method, the LDH is added to a solution containing the desired anion, heated, and stirred. The anion can be organic or inorganic. The anions can then diffuse in and out of the LDH interlayer because the LDH structure favours anion diffusion, exchanging the original anions with the desired anions. The diffusion of the in-going anions within the interlayer is the rate-determining step of the process [18]. In-going anions with a high charge density are favoured during exchange [14]. NO${}_{3}^{-}$ or Cl${}^{-}$ have low charge densities and for this reason LDHs containing these anions are good precursors for exchange [31].

The reconstruction technique utilises the structural memory property of LDH. This property allows the reconstruction of an LDH structure by hydrating an LDH that was calcined into a mixture of metal oxides [14]. This method can be used to modify the interlayer anion. First, the precursor anion is removed during calcination, then the calcined LDH is rehydrated in an aqueous solution containing the desired anion [43].

LDH containing an organic interlayer anion can also be synthesised in a one-step method. In this method, the metal salt solution is added dropwise to an aqueous solution containing the desired anion at a controlled pH [44]. This method simplifies the process by removing a second step of anion exchange or reconstruction.

## 4. Crosslinking of Elastomers

During crosslinking, a three-dimensional structure is formed in the rubber matrix. This drastically improves the mechanical properties of the elastomer. The crosslink density affects the mechanical properties and must be controlled so that the desired properties are achieved. Crosslinks can consist of a group of sulphur atoms, a single sulphur atom, carbon–carbon bonds, a polyvalent organic radical, ionic cluster or a polyvalent metal ion [45].

### 4.1. Sulphur Vulcanisation

Sulphur vulcanisation is a widely used method that can be used to crosslink natural rubber (NR), SBR, NBR, EPDM, and many others [45]. Sulphur vulcanisation works for elastomers that have accessible double bonds. It requires several different reagents, namely vulcanisation agents, accelerators, activators, retarders, and prevulcanisation inhibitors. Vulcanisation agents provide the sulphur used during the crosslinking process and can be either elemental sulphur or an organic sulphur donor compound such as tetramethylthiuram (TMTD) or dithiodimorpholine (DTDM). Replacing the elemental sulphur in part or completely with sulphur donors improves thermal and oxidative degradation resistance and reduces sulphur bloom [46].

There are various classes of accelerators and they affect the crosslinking process in different ways. Some are faster than others. Important accelerator classes are sulfenamides, benzothiazoles, guanadines, and dithiocarbamic acid. Metal oxides (especially ZnO), fatty acids such as stearic acid, and nitrogen-containing bases are used as activators [45]. In order to prevent premature vulcanisation, retarders and prevulcanisation inhibitors such as n-cyclohexylthiophthalimide (CTP) are also added to the rubber [47].

The mechanism of vulcanisation is still not fully understood and there are different pathways still being disputed. Both ionic pathways and free radical pathways have been proposed [45,47]. There is still much uncertainty about the nature of the active sulphurating agent and its reaction with rubber [47].

The accepted general reaction path of sulphur vulcanisation starts with the accelerator reacting with sulphur to give monomeric polysulphides. These polysulphides interact with the rubber to form polymeric polysulphides with the rubber. The rubber polysulphides then react to give crosslinks where the sulphur connects two rubber chains [45].

### 4.2. Crosslinking through Reaction with Metal Oxides

Polar rubbers such as CR and XNBR can be crosslinked using metal oxides. ZnO is most commonly used as the crosslinking agent, and can be used alone, but MgO is added to provide scorch resistance. In CR, it is thought that the allylic chlorine atom reacts with the metal oxide. The reaction of the rubber with the ZnO results in the formation of carboxylic salts that act as ionic crosslinks [17]. Sulphur is also added to the rubber to increase the cure rate, although CR does not cure in the same way as NR, butadiene rubber (BR), SBR, etc. Ethylene thiourea (ETU) is usually used as the accelerator, but a combination of TMTM/DOTG/sulfur is also sometimes used because it has better scorch resistance properties, at the cost of cure rate [48]. Due to the fact that ETU is a suspected carcinogen, alternative accelerators, such as thiocarbanalide, are being considered [45].

According to Ibarra et al. [49], the salt crosslinks that are formed provide high tensile strength, tear and abrasion resistance, hardness and self-reinforcement. However, vulcanisates crosslinked using metal oxides tend to be unstable under stress and high hysteresis.

### 4.3. Organic Peroxide Crosslinking

Peroxide crosslinking can be used to vulcanise many elastomers, such as SBR, NBR, XNBR, EPDM, SR and PU. The peroxides are decomposed to form radicals that can then react with the polymer, forming a new radical that can react with a radical on another chain, forming the crosslinks. In unsaturated polymers such as SBR and NBR, the radicals will attack the double bonds. The organic peroxides used include dialkyl peroxides, t-butyl perbenzoate, dicumyl peroxide, 1,1-bis(t-butylperoxy)-3,3,5-trimethylcyclohexane and 2,5-dimethyl-2,5-bis(t-butylperoxy)hexane [50].

As stated by Coran [45], when a saturated hydrocarbon elastomer is crosslinked using peroxides, the mechanism is slightly different. The peroxide is decomposed and the radical then reacts with a hydrogen atom on the polymer chain, leaving a radical on the chain that can form a crosslink with another polymer chain with a radical. Branches will decrease the crosslinking efficiency because the peroxide can be used up by chain scission before enough crosslinks are formed. Adding sulphur and other coagents can reduce the chain scission reactions. Some coagents have the added benefit of reducing scorch, which is a problem in peroxide crosslinking. Acidic compounding additives such as fatty acids, some carbon blacks and acidic silicas can cause unwanted decomposition of peroxides because they act as catalysts. Other additives can reduce the crosslinking efficiency because they quench or alter the free radicals needed to react with the polymer.

## 5. Layered Double Hydroxides in Rubber

**Figure 7:**
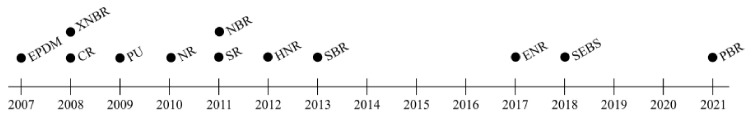
Order in which the first articles on each elastomer were published.

In this section the use of LDH in different types of elastomers is discussed. The elastomers are discussed in order of appearance in the literature search results. The first elastomer found in the literature search was EPDM in 2007, for example, and as such is the first elastomer that is discussed. Figure 7 shows the order in which the first article on each elastomer was published.

In some studies two types of elastomers were blended together in a 50:50 wt % ratio. In the case of these polymer blends, the articles are discussed in the section of the elastomer that was first in the publication order and only mentioned in the section of the other elastomer in the blend.

The properties that were typically looked at when investigating the effect of LDH on the properties of rubber include tensile strength (TS), elongation at break (EB) and modulus. The modulus values can be reported at different elongation values, typically at 100% elongation (M100), 200% elongation (M200) or 300% elongation (M300). Other properties that were looked at are the glass transition temperature (T${}_{g}$) and the tan(δ) peak height. The tan(δ) peak height gives an indication of chain mobility.

### 5.1. Ethylene Propylene Diene Monomer Rubber (EPDM)

EPDM contains some double bonds on the side groups. It is resistant to ozone attack, oxidation and moisture, making it useful for applications where weather and heat resistance is required [3]. EPDM can be crosslinked using sulphur vulcanisation and peroxide crosslinking [51].

Of the 61 articles found during the literature search, 6 articles were on the use of LDH in EPDM. The first two articles were published in 2007 and then one per year was published in 2008, 2009, 2016 and 2020. Of the six articles, four used Mg-Al LDH and two used Zn-Al LDH. A total of five of the six LDHs were organically modified.

In two studies, Mg-Al LDH was modified with dodecyl sulphate (DS-LDH) and was added to EPDM at different loadings. The increase in interlayer spacing caused by the organic modification allowed the LDH layers to become partially exfoliated and the increased compatibility with EPDM, causing the LDH to be well dispersed [52,53]. The exfoliation of the LDH increased the polymer–filler interaction. Increasing the DS-LDH content increased the TS and EB of the composites. The interfacial interaction between the DS-LDH and EPDM allowed efficient stress transfer from the polymer, enhancing the TS and increasing the stiffness of the composite. The platelet orientation and chain slippage increased the EB. The dodecyl sulphate chain in the LDH may also have had a plasticising effect that increased EB. The modulus also increased due to the polymer–filler interaction and the sterically hindered LDH surface creating resistance. DS-LDH platelets altered the crack propagation in the EPDM because they acted as obstacles to crack propagation and caused cracks to branch off. At higher concentrations of LDH, the mechanical properties started to decrease again due to the presence of agglomerates. The thermal stability of the EPDM increased, reached a maximum and decreased again with increasing DS-LDH content This is similar to what was observed for the mechanical properties due to agglomeration. The exfoliated DS-LDH layers could act as barriers to volatile decomposition products and heat, improving thermal stability. T${}_{g}$ was not affected by the addition of DS-LDH, which showed that the polymer chains did not become entangled around the LDH particles.

The effect of matrix polarity and functional groups on the dispersion of LDH, the polymer–filler interaction and the composite mechanical properties was investigated by Pradhan et al. [13]. They compared EPDM, a non-polar rubber, to XNBR, a polar rubber with carboxyl functional groups that can interact with the hydroxyl groups on LDH. The XNBR results are discussed in detail in the section on XNBR. The Mg-Al LDH was modified with sodium 1-decanesulphonate (C10-LDH). EPDM did not interact with the C10-LDH during melt compounding and there was no intercalation or exfoliation observed. In EPDM, the C10-LDH was mostly present as individual particles and aggregates. The storage modulus of the EPDM composites decreased and then increased with increasing C10-LDH content. At low loadings the plasticising effect of the organic modifier dominated over the low reinforcing effect of the LDH due to poor polymer–filler interaction. At higher loadings the reinforcing effect of LDH increased and dominated over the plasticising effect. TS, modulus and EB increased with increasing C10-LDH content, similarly to what was observed by Acharya et al. [52]. The tan(δ) peak was not affected by the addition of C10-LDH, which showed that the interaction between the EPDM and C10-LDH was weak. Microvoids formed around the LDH particles, which also showed the interaction was weak. The formation of microvoids does, however, absorb energy during deformation which could help improve the mechanical properties of the composites.

Kuila et al. [54] investigated the effect of dodecyl sulphate-modified Mg-Al LDH (DS-LDH) in a 50:50 wt % EVA/EPDM blend. The EVA used had a 45 wt % VA content. The DS-LDH was homogeneously distributed and exfoliated at low loadings, but agglomeration occurred at higher loadings. There was good interfacial interaction between DS-LDH and the polymer blend. The LDH hydroxyl groups could interact with the polar acetate groups in the EVA, which improved the polymer–filler compatibility and interaction. This caused the TS to increase as the DS-LDH concentration was increased. The EB was also improved due to the plasticising effect of the organic modifier, chain slippage and platelet orientation. The storage modulus was increased and the tan(δ) peak was decreased by the polymer–filler interaction restricting chain movement and making the composite more stiff. The T${}_{g}$ was unaffected by the DS-LDH. The thermal stability increased as the DS-LDH loading increased due to the barrier effect of the platelets and the char formation caused by the decomposition of the LDH. Agglomeration at higher loadings caused a decrease in thermal stability. Increasing the DS-LDH concentration first decreased and then increased the crosslink density. This may have been caused by the LDH platelets becoming more ordered and gradually adsorbing less of the peroxide crosslinker as the LDH content of the composite was increased. It can be concluded that the exfoliated DS-LDH platelets can act as a reinforcing filler and enhance the mechanical properties of the polymer blend, although agglomeration at higher LDH concentrations still poses a challenge.

Basu et al. [55] reported successfully adding up to 100 phr (parts by weight per hundred resin) unmodified Zn-Al LDH to EPDM and found that crosslinking took place even in the absence of ZnO. The Zn ions of the LDH could therefore participate in the curing reaction. At 10 phr and above, the LDH was more efficient at curing the EPDM than ZnO. The TS and EB were increased compared to the ZnO-cured EPDM. The maximum increase was at 10 phr LDH before decreasing again. The modulus continued to increase with increasing LDH content. T${}_{g}$ was also increased by adding LDH which shows that there is good interaction between the LDH and rubber despite the LDH being unmodified.

EPDM can be blended with polypropylene (PP) in the presence of LDH to form a dynamically vulcanised thermoplastic elastomer. Vol’fson and Nikiforov [56] added stearate-modified Zn-Al LDH to a 60:40 weight ratio EPDM/PP blend. They found that the stearate modification improved compatibility between the LDH and polymer blend. Due to this the TS and EB were improved, although agglomeration decreased the mechanical properties at higher LDH loadings. The LDH also increased the thermal stability of the composites. The barrier effect of the LDH platelets, as well as the endothermic degradation of the LDH helped to form a char layer that prevented the diffusion of volatile degradation products and oxygen.

The effects of LDH on the mechanical properties on EPDM are summarised in Table 1. The percentage change in each property compared to neat EPDM is shown. In some cases the data were only shown graphically and as such these percentages should only be used as an indication of the influence of LDH on the properties of the rubber and not as absolute values. In cases where multiple loadings of the same LDH were tested, the loading with the highest TS was used in reporting the mechanical properties. This method was used for summary tables for all sections where data were available. Due to the fact that experimental conditions varied from study to study, comparisons between results are difficult to make. The summary tables should therefore be viewed as a summary of work conducted so far rather than a direct comparison of results. The study by Acharya et al. [53] was omitted as mechanical property values were not discussed. An approximation of the wt % loading of LDH in the study by Vol’fson and Nikiforov [56] was made as limited data were available.

### 5.2. Carboxylated Nitrile Rubber (XNBR)

According to Thakur et al. [57], XNBR is a terpolymer containing acrylonitrile, butadiene and monomers that have carboxyl groups such as acrylic acid and methacrylic acid. The pendant carboxyl groups can react with curing agents and provide additional curing sites. The carboxyl chains improve the intermolecular and intramolecular interactions between the chains, which leads to improved TS and loss of extension and recovery properties. While the mechanical properties of XNBR are good, the double bonds in the backbone of the rubber cause it to be sensitive to ozone and UV light [58]. XNBR can be crosslinked using inorganic peroxides such as zinc peroxide [47] and through ionic cluster crosslinking via metal oxides such as ZnO [59]. A mixed system of zinc peroxide and sulphur accelerators can also be used [60].

**Figure 8:**
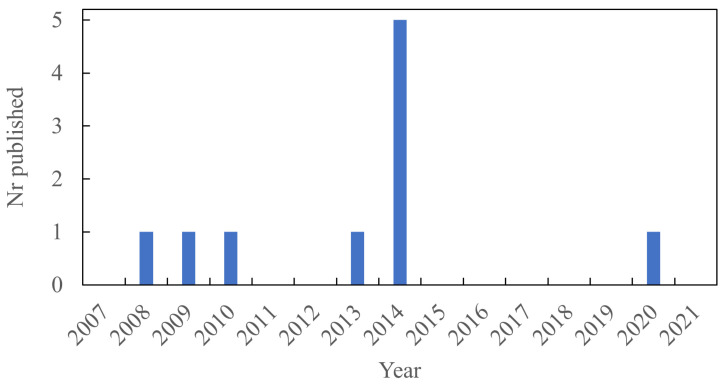
Number of articles published on LDH in XNBR per year.

XNBR was used in 10 of the articles. Figure 8 shows the distribution of papers published over the years. In the majority of these studies, unmodified Mg-Al LDH was used. One study used Zn-Al LDH and in two studies the LDH was modified with 1-decanesulphonate or 1-hexadecanesulphonate.

In the study conducted by Pradhan et al. [13] where XNBR was compared to EPDM, there was evidence that the 1-decanesulphonate-modified Mg-Al LDH (C10-LDH) interacted with XNBR during melt compounding. FTIR showed that reactions took place between the LDH hydroxyl groups and the XNBR carboxyl groups. XNBR chains were intercalated into the LDH interlayer. The polar nature of XNBR may have made the intercalation easier when compared to nonpolar EPDM. Exfoliation also occurred, most likely due to the strong interaction between the LDH and XNBR causing the LDH layers to be peeled apart during shear mixing. There was minimal agglomeration of C10-LDH particles in XNBR. The storage modulus and TS increased with increasing LDH content while EB decreased. This is due to the strong polymer–filler interaction. The C10-LDH increased the strain-induced crystallisation of XNBR, similar to what happens when conventional reinforcing fillers such as carbon black are used. The exfoliated particles had large surface areas and this, combined with the strong polymer–filler interaction, facilitated orientation of the XNBR chains and initiated crystallisation at lower strain. Additional crosslinks could also form due to the chemical interaction between the polar groups of the LDH and XNBR, showing that LDH can act as both a reinforcing filler and a crosslinking agent in XNBR.

Thakur et al. [57] further investigated the effect of LDH on the cure and mechanical properties of XNBR composites containing ZnO. In order to minimise the effect of other interactions, no other crosslinking agents were added. The Mg-Al LDH was modified with sodium dodecylbenzenesulphonate (DBS-LDH). During melt compounding the organically modified LDH was shown to have greater interaction with XNBR than the unmodified LDH. While LDH has hydroxyl groups that can interact with the polar groups of XNBR, the organic modification further improved interaction by making it more hydrophobic and therefore more compatible with the hydrocarbon chains. The interlayer spacing of the LDH was also increased by the organic modifier, making it easier for polymer chains to intercalate into the LDH. The modified LDH was dispersed better in the rubber matrix than unmodified LDH and was also partially exfoliated, although there were still some agglomerates. The XNBR filled only with ZnO underwent self crosslinking through the formation of anhydride bridges. When unmodified and modified LDH was added to the XNBR, this self crosslinking was no longer observed. This indicated that the LDH may have suppressed the formation of anhydride linkages. LDH also suppressed the formation of ionic crosslinks between XNBR and ZnO. The authors suggested that this may be due to the hydroxyl groups of LDH forming coordination complexes with the zinc ions, leaving some of the carboxyl groups free in the XNBR. The organically modified LDH suppressed the ionic crosslinking to a lesser degree because the modifier prevented the LDH from entering into the coordination complex. The TS was significantly decreased by the unmodified and modified LDH due to the suppressed ionic crosslinking. The results of this work showed that LDH can interact with the crosslinking system and acts as an ionic crosslink inhibitor in XNBR. This negatively impacts the mechanical properties of the composites. Modification of the LDH reduced the inhibition but did not completely prevent the inhibition.

As shown previously, LDH can participate in the curing reactions and also affect the strain-induced crystallisation behaviour. Costa et al. [59] investigated the influence of the organic modifier and dispersion of the LDH on the strain-induced crystallisation. Mg-Al LDH was modified with sodium 1-decanesulphonate (C10-LDH) and sodium 1-hexadecanesulphonate (C16-LDH). C16-LDH had a larger interlayer spacing than C10-LDH. As observed in previous work, the basic nature of LDH allowed it to be reactive to XNBR and participate in the crosslinking process. A very small amount of ZnO was added so that it would not significantly affect the crosslinking or interact with the organic modifiers of the LDH. The chemical interaction between the LDH and XNBR could be seen in the increase in TS of the composites. The type of modifier also had an influence on the properties of the XNBR. Before vulcanisation, C10-LDH was not dispersed as well as C16-LDH. During vulcanisation, while tactoids of both LDHs grew larger, no new C10-LDH tactoids formed while new C16-LDH tactoids formed, possibly due to exfoliated layers collapsing. This caused C16-LDH to be more poorly dispersed and have fewer exfoliated layers. While a larger interlayer spacing usually leads to more exfoliation and polymer chain intercalation, the changes during vulcanisation caused the opposite to occur. Both LDHs interacted with XNBR and caused an improvement in TS, but C10-LDH caused a greater improvement. The difference in TS was greater at higher concentrations of LDH. The C10-LDH increased strain-induced crystallisation while C16-LDH did not due to the large tactoids restricting chain movement and thus preventing the chains from aligning. The nature of the organic modifier on the can LDH therefore have a significant influence on the final properties of the XNBR composites.

Laskowska et al. [61] investigated the effect of two ionic liquids (ILs) on the curing behaviour and mechanical properties of XNBR crosslinked with Mg-Al LDH, and NBR reinforced with silica and crosslinked using a sulphur cure system. The ILs used were hydrophilic 1-ethyl-3-methylimidazoliumthiocyanate (EMIM SCN) and hydrophobic 1-ethyl-3-methy-limidazolium bis(trifluoromethylsulfonyl)imide (EMIM TFSI). At 5 phr, EMIM SCN increased the stiffness and torque of both rubbers. At higher concentrations, the EMIM SCN reduced the torque. EMIM TFSI reduced the torque at all concentrations. This showed that the ILs had a negative effect on the formation of polar bonds between the LDH and XNBR as well as on the formation of covalent bonds in the NBR/SiO${}_{2}$ composites. At 5 phr both ILs increased the rate of cure. The hydrophilic EMIM SCN was more effective because it better assisted in the dissolution and diffusion of crosslinking agents in the rubber. Further increasing the IL concentration decreased the rate of cure for both rubbers. Initially EMIM TFSI decreased the scorch time (t${}_{2}$) and optimum cure time (t${}_{90}$) but further increased the concentration decreased the rate of cure. EMIM SCN decreased the t${}_{90}$ of the NBR/SiO${}_{2}$ composites but not of the XNBR/LDH composites. A value of 5 phr EMIM SCN increased the modulus of both rubber composites but a further increase in IL concentration reduced the modulus. EMIM TFSI decreased the modulus at all concentrations. EMIM SCN increased the TS at 5 phr and then decreased the TS at higher concentrations for both rubbers. It decreased the EB at all concentrations. EMIM TSFI increased the TS and EB at all concentrations. At 5 phr both ILs increased the crosslink densities but the crosslink densities decreased with increasing IL content. EMIM SCN did not affect the T${}_{g}$ of the XNBR composites and EMIM TFSI lowered the T${}_{g}$ with increasing IL content due to a plasticising effect. In the case of NBR, EMIM SCN decreased the T${}_{g}$ slightly with increasing IL content but EMIM TFSI did not have any effect. The plasticising effect may have been inhibited by the silica adsorbing the IL. This did not occur in the composites containing LDH. Hydrophobic EMIM TFSI improved dispersion of the LDH and silica. The authors speculate that this could be due to the fillers being coated in a layer of IL as well as adsorption of the IL, which decreased the polarity of the filler surfaces and increased compatibility with the rubber.

Marzec et al. [62] continued the work on using ILs in XNBR crosslinked with Mg-Al LDH. They used two ILs, namely 1-butyl-3-methylimidazolium bis(trifluorome-thylsulfonyl)imide (BMIM TFSI) and 1-butyl-3-methylimidazolium tetrachloroaluminate (BMIM AlCl${}_{4}$) at various concentrations. The first is hydrophobic and the second is hydrophilic. BMIM AlCl${}_{4}$ increased the crosslink rate and decreased the scorch time and optimum cure time even at a relatively high loading of 15 phr. BMIM TFSI did not seem to affect the curing characteristics of the XNBR. BMIM AlCl${}_{4}$ also increased the crosslink density of the XNBR with increasing IL content while BMIM TFSI decreased it. This was due to the BMIM TFSI being adsorbed onto the LDH surface, decreasing the interactions between the LDH and rubber. BMIM AlCl${}_{4}$ decreased TS and EB with increasing IL content and increased the modulus at 2.5 phr but decreased it again at higher concentrations of IL. This is due to the high crosslink density and reduced mobility of the polymer chains. On the other hand, the TS and EB of the BMIM TFSI composites increased, reached a maximum and then decreased with increasing IL content. All modulus values where BMIM TFSI was used were lower than for the neat XNBR/LDH composite. BMIM TFSI acted as a plasticising agent and did not affect the crosslink structure, which is why it had this effect on the mechanical properties. The plasticising effect also caused a decrease in the T${}_{g}$ and the storage modulus. BMIM AlCl${}_{4}$ did not affect the T${}_{g}$ as much, although it did lower the T${}_{g}$ somewhat.

This research group also compared the effect of EMIM TFSI, BMIM TFSI and 1-hexyl-3-methylimidazolium bis(trifluoro-methylsulfonyl)imide (HMIM TFSI) on the properties of XNBR cured with Mg-Al LDH [63]. Adding ILs decreased the t${}_{2}$ and t${}_{90}$ values and increased the cure rate index (CRI) values compared to when no IL was used. The TFSI anions therefore accelerated the curing process. The amount and type of IL did not have a significant effect. HMIM TFSI had the greatest plasticising effect in the rubber because it has the longest alkyl chain, and decreased the viscosity of the uncured rubber. At low concentrations the EMIM TFSI and BMIM TFSI slightly increased EB and TS but as the IL concentration was increased the TS decreased. The plasticising effect of HMIM TFSI decreased the TS compared to neat XNBR/LDH at all concentrations. The ILs decreased the crosslink density of the rubber, which showed that they hindered the rubber–filler interactions that formed the ionic crosslinks. EMIM TFSI had the smallest negative impact on the properties. The T${}_{g}$ was decreased by all three ILs due to their plasticising effect, and the decrease in T${}_{g}$ was largest for the IL with the longest alkyl chain (HMIM TFSI).

Laskowska et al. [58] continued their work on using unmodified Mg-Al LDH in XNBR and showed that LDH can be an effective ionic crosslinking agent for XNBR in the place of ZnO. They showed that ionic bonds formed when LDH was used without any ZnO present and that the crosslinking density increased with increasing LDH content. At low concentrations the LDH cure resulted in a lower crosslink density than ZnO, but the 10 phr LDH composite had a higher crosslink density than the 5 phr ZnO composite. The LDH-cured XNBR composites had lower scorch times and CRIs than ZnO-cured XNBR. Because the crosslink density increased with increasing LDH content and the XNBR and LDH had strong polar interactions, the T${}_{g}$, TS and storage modulus increased while EB decreased in comparison to neat XNBR. The ZnO-cured composite had lower EB and higher TS values than any of the LDH-cured composites but LDH improved the tear strength more than ZnO at higher loadings. An ionic transition, associated with the formation of ionic clusters, was observed for all the composites in the DMA results. The LDH composites had lower ionic transition temperatures than the ZnO composite. LDH can clearly be used as both a reinforcing filler and a crosslinking agent in XNBR.

Laskowska et al. [64] found that the Mg content, aspect ratio and surface area of the LDH have an effect on the properties of XNBR. They added unmodified Mg-Al LDH with varying Mg:Al ratios and aspect ratios to XNBR and found that increasing the Mg content increased the cure efficiency, number of ionic bonds in the rubber, crosslink density, TS and storage modulus. A larger surface area and aspect ratio also increased the curing efficiency due to the increased interaction between the LDH and XNBR. This increased interaction also led to an increase in mechanical properties, although it also decreased EB. As shown previously, LDH can crosslink XNBR [13], which is why the Mg content and interaction affects curing efficiency. Adding LDH to the XNBR increased the T${}_{g}$ but the Mg:Al ratio and filler loading did not affect how much the T${}_{g}$ was shifted.

In ionic elastomers such as XNBR, the ionic clusters formed during crosslinking affect the mechanical properties of the rubber. The clusters exist as a large number of ion pairs as well as hydrocarbons [65]. ZnO can be used as an ionic activator but the use of ZnO results in scorchiness, poor flexibility and high compression set [49]. Basu et al. [17] compared Zn-Al LDH, ZnO and ZnCl${}_{2}$ in metal oxide and sulphur cures and investigated the nature of the ionic interactions in XNBR. They found that in the metal oxide cure these Zn-containing compounds all formed crosslinks through forming coordination complexes. The ZnO-cured compound was the only one to show a second, high temperature transition. The authors proposed that the ZnO reacted with carboxylate groups and formed a three-dimensional polymeric network through formation of coordination complexes. The crosslinked network could then act as a separate phase with its own T${}_{g}$. The LDH and ZnCl${}_{2}$ did not coordinate to form a polymeric network structure. In the sulphur cure, LDH was more efficient than ZnO at equivalent concentrations of Zn, resulting in higher crosslink density.

It was previously shown that the Mg:Al ratio influenced the properties of XNBR [64] and the effect of the Mg:Al ratio and surface area of LDH on the properties of XNBR was further investigated by Lipińska et al. [66]. In XNBR cured only with LDH, the optimal curing time and the onset curing temperature increased as the Al content of the LDH was increased. Increasing the surface area of the LDHs increased the availability of hydroxyl groups and cation sites, which increased the rate of cure. Curing could take place through polar interactions between XNBR and LDH as well as through the formation of ionic crosslinks between the LDH metal ions and carboxylic groups of XNBR. This is why an increased Mg content increased the curing efficiency through promoting the formation of the more ordered ionic phase. Increasing the Mg content of the LDH increased the polymer–filler interfacial interaction and decreased the filler–filler interactions.

Table 2 summarises the effect of the LDHs on the properties of XNBR. In cases where more than one loading was used, the loading that resulted in the highest TS was used as the point of comparison and where different types of LDHs were used, all types are shown in the table.

### 5.3. Chloroprene Rubber (CR)

CR is another diene-based polymer, containing chlorine and double bonds on the backbone chain. It is also known as neoprene [67]. CR is resistant to ozone and UV degradation and, due to the chlorine content, is more resistant to burning than rubbers that contain only hydrocarbons [68]. It is also tough and useable over a wide range of temperatures [67]. CR is normally crosslinked using metal oxides, usually ZnO with added MgO to improve scorch resistance. The metal oxide can also be mixed with a sulphur accelerator [45].

Only one article, published in 2008, considered the effect of LDH on the mechanical properties of CR.

The effect of LDH and MMT on the properties of CR was investigated by Das et al. [69] and compared to a reference composite containing carbon black. Modified and unmodified Mg-Al LDH and MMT were tested. The LDH was modified with dodecylbenzenesulphonate (DBS-LDH) and the MMT was modified with a quaternary ammonium salt (O-MMT). Both clays could undergo hydrogen bonding with the ethylene thiourea (ETU) used as an accelerator, which slowed the curing process and thus reduced the CRI. Carbon black, LDH and MMT all interfered with the curing process and reduced the CRI. Unmodified LDH caused the greatest reduction because it interacted more strongly with the ETU. Organic modification of the LDH increased the cure rate, whereas the cationic modifier on MMT caused a further reduction in the cure rate. The modulus values increased with addition of all three fillers. The nature of the clay and organic modifier both played a role in enhancing the mechanical properties of CR. Unmodified LDH improved the modulus more than unmodified MMT, which had a similar modulus value to carbon black. O-MMT caused a more significant change in modulus values than DBS-LDH. DBS-LDH had a lower organic content than O-MMT. The organic modifiers had a plasticising effect that facilitated chain slippage and therefore enhanced the mechanical properties such as TS and EB. O-MMT increased the storage and loss modulus the most and DBS-LDH gave the second largest increase. Overall O-MMT was a better reinforcing filler than DBS-LDH in CR. Modified and unmodified MMT also enhanced the crystallisation of CR whereas LDH and carbon black suppressed crystallisation. This study shows that the nature of the layered clay as well as the organic modification affects the properties of chloroprene rubber.

Table 3 shows how the two types of LDH affected the properties of CR.

### 5.4. Polyurethane (PU)

PUs are polymers that have urethane groups and can thus form hydrogen bonds. PU elastomers are a subset of PU polymers. They have high chemical abrasion resistance and have good mechanical properties [70]. PU elastomers have soft and hard segments. The soft segments provide most of the elastomeric properties and the hard segments provide tie points that act as crosslinks and as reinforcing fillers [2]. PU can be crosslinked using peroxide crosslinking [45].

**Figure 9:**
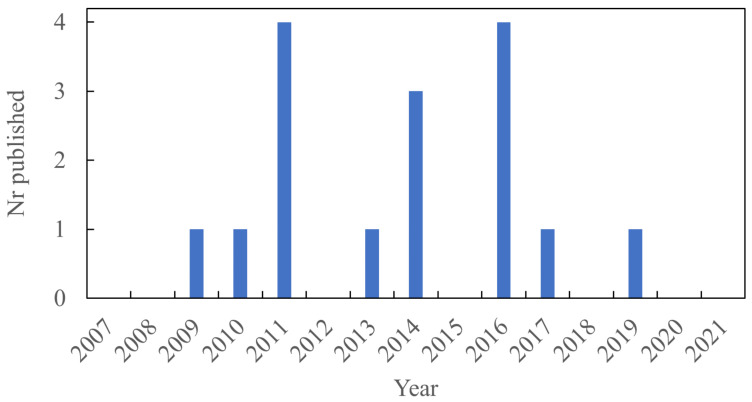
Number of papers published on LDH in PU per year.

There are 16 papers published on the effect of LDH on PU that fit the criteria of the systematic literature review. Figure 9 shows the distribution of papers published.

Dodecyl sulphate-modified Mg-Al LDH (DS-LDH) was added to PU by Kotal et al. [71] to investigate the effect of LDH on the properties of the rubber. The DS-LDH was intercalated and partially exfoliated in the polymer matrix. The hydroxyl groups of DS-LDH interacted with the polar groups of PU. This strong interfacial interaction and exfoliation enhanced stress transfer and increased the TS as the DS-LDH concentration was increased. The EB increased because of the the polymer chains becoming entangled as well as the synergy between chain slippage and DS-LDH platelet orientation. The DS-LDH particles acted as barriers to crack propagation, causing secondary cracks to form at the rubber/filler interface. During deformation, microvoids formed around the DS-LDH particles. The microvoid formation absorbed energy and enhanced the mechanical properties. DS-LDH also improved the thermal stability of PU. The mechanical properties and thermal stability increased, reached a maximum and then decreased as the DS-LDH concentration was increased. This is because agglomerates formed at higher concentrations of DS-LDH that weakened the polymer–filler interaction. Organically modified LDH can therefore be used to enhance the mechanical properties and thermal stability of PU, although when the LDH loading becomes too high the reinforcing and stabilising effect is reduced due to agglomeration.

Kotal et al. [41] prepared 50:50 weight ratio PU/NBR blends containing Mg-Al LDH-modified with dodecyl sulphate (DS-LDH) at different loadings. Partial exfoliation and polymer chain intercalation took place, although agglomeration occurred at higher loadings of DS-LDH. The LDH hydroxyl groups could interact strongly with the polar groups of PU and NBR. This improved interfacial adhesion which, combined with good dispersion, enabled efficient stress transfer and increased the stiffness of the composites. This in turn improved the TS of the composites. EB was improved by the polymer chains becoming entangled around the DS-LDH platelets through chain slippage and platelet orientation. This improvement in mechanical properties was, however, only observed at the lowest loading of DS-LDH. As the DS-LDH loading was increased, the mechanical properties started to decrease. This was most likely caused by the presence of agglomerates at higher DS-LDH loadings that weakened the rubber–filler interaction and promoted crack growth. The storage and loss moduli increased, reached a maximum and then decreased again with increasing DS-LDH content. The increase in storage modulus was due to the strong rubber–filler interactions restricting polymer chain movement, while the increase in loss modulus was due to the dispersion and high aspect ratio of the DS-LDH platelets increasing the friction between the rubber and filler. The restricted chain movement also caused an increase in T${}_{g}$ at lower LDH concentrations. DS-LDH also increased the thermal stability of the polymer blend, similarly to what has been observed in previous studies.

The effect of dodecyl sulphate-modified Mg-Al LDH (DS-LDH) on the properties of PU was investigated further by Kotal and Srivastava [72], particularly the effect on shear and peel strength. At low loadings the LDH was partially exfoliated and at higher levels agglomeration occurred and dispersion was poor. Their previous work showed that the improvement in TS and EB reached an optimum [71]. The DS-LDH made the composites more stiff, increasing the storage modulus. The T${}_{g}$ was increased by adding DS-LDH due to the interaction between PU and LDH restricting polymer chain movement. The storage modulus and T${}_{g}$ decreased again at higher loadings of LDH. The lap shear strength and peel strength were both increased by addition of DS-LDH. The adhesive strength of the PU was increased by the improved lap shear and peel strength as well as the strong interaction between the LDH and PU. At higher loadings of LDH the improvement in the properties was less due to the presence of agglomerates.

Kotal et al. [73] did another study using PU, but modified the Mg-Al LDH with sodium stearate (St-LDH). At low loadings, the St-LDH layers were exfoliated, but at higher levels polymer chains intercalated into the St-LDH layers and distribution became inhomogeneous. The St-LDH hydroxyl groups underwent hydrogen bonding with the PU. The authors also predicted whether the mixing of LDH and PU is favourable by using the thermodynamic relationship in Equation (1) for PU and St-LDH [74].

(1) $$\begin{array}{cc} \Delta G & =\Delta H-T\Delta S \end{array}$$

The mixing of LDH and PU was determined to be favourable because the total change in Gibbs free energy, as given by Equation (2), was negative at 8 wt % St-LDH.

(2) $$\begin{array}{cc} \Delta G_{S} & =\Delta G_{PU}+\Delta G_{LDH} \end{array}$$

The mechanical properties of PU were improved by the addition of St-LDH. The increase in TS and modulus were at a maximum at the lowest LDH loading and decreased with increasing LDH content. At the lowest loading, 1 wt % St-LDH, the exfoliation was best, which may explain why the reinforcement was best at this loading. The high degree of exfoliation and large aspect ratio of the LDH particles enhanced the interfacial interaction between the DS-LDH and PU via hydrogen bonding. As the St-LDH loading was increased, the filler–filler interaction became greater and agglomerates formed, thus decreasing the interaction between LDH and PU and lowering the TS. The high aspect ratio as well as the development of shear zones was most likely responsible for the increase in modulus. The EB was also increased by St-LDH. The increase reached a maximum at a higher concentration than the TS. The bonding between St-LDH and PU enabled the composite to resist breaking when it was extended, but the improvement in EB may also have been due to the stearate anion having a plasticising effect caused by its long alkyl chain. At higher loadings where the EB started to decrease, the agglomerated St-LDH particles restricted chain movement and thus the shear deformation of the PU was reduced. This led to a decrease in EB. In comparison to the previous work performed with DS-LDH [71], the improvement in EB was larger for St-LDH. This may be because the stearate anion’s longer hydrocarbon chain gave the matrix more flexibility. This may also have caused the decrease in modulus values. The T${}_{g}$ of the composites increased with increasing St-LDH content due to the restricted chain movement. The storage and loss moduli also increased with increasing St-LDH content due to increased friction between the filler and polymer as well as the restricted chain movement. This restricted mobility of the polymer chains at the polymer filler interface also reduced the height of the tan(δ) peak and broadened it. St-LDH also proved to be a good thermal stabiliser for PU. The barrier effect of the exfoliated St-LDH layers as well as the endothermic decomposition of LDH helped to increase the thermal stability of the composite.

In the same year, Kotal and Srivastava [75] found that organically modified Mg-Al LDH had a syngergistic effect with methylenediphenylene diisocyanate (MDI) and isophorone diisocyanate (IPDI) in PU. MDI and IPDI were successfully grafted into stearate and dodecyl sulphate-modified LDH (St-LDH and DS-LDH). The organic modification of the LDH increased the interlayer spacing of the LDH and allowed the MDI and IPDI to enter the interlayer and react with the hydroxyl groups of the LDH. The intercalation of MDI and IPDI did not occur when the LDH was unmodified, which showed the importance of increasing the interlayer spacing through organic modification. The modified MDI-LDH and IPDI-LDH were uniformly dispersed in the PU matrix and were exfoliated. The unmodified MDI-LDH and IPDI-LDH formed agglomerates. The MDI-St-LDH dispersed best and had the most exfoliation. There was also evidence of hydrogen bonding between the PU and modified and unmodified LDHs. The modified MDI-LDH and IPDI-LDH composites improved the TS more than unmodified MDI-LDH and IPDI-LDH or DS-LDH and St-LDH. This shows that the organic modifiers and MDI and IPDI have a synergistic effect. The stearate-modified MDI-LDH and IPDI-LDH composites had the largest increase in TS due to the chemical bonding between the isocyanate grafted St-LDH and PU. The MDI-grafted LDH composites had a smaller improvement in EB than their IPDI counterparts. The MDI-grafted LDHs restricted chain movement more than the IPDI-grafted LDHs, which increased the stiffness of the composite and decreased EB. The MDI-St-LDH composite had the highest crosslink density and the authors suggest that this is due to the MDI being more reactive than IPDI. All LDH composites had a higher thermal stability than neat PU but the greatest increase was in the MDI-St-LDH composite. This composite had good dispersion, which means the barrier effect of the LDH was effective. The T${}_{g}$ of the composites containing MDI and IPDI grafted LDH increased. The stearate-modified MDI-LDH had the greatest increase in T${}_{g}$. The interaction between the filler and polymer restricted chain movement which increased the T${}_{g}$, showing that MDI-St-LDH had the best interaction with PU.

Guo et al. [76] found that adding dodecyl sulphate-modified Co-Al LDH to PU improved the mechanical properties and improved the flame retardancy of the composite. At low loadings, the LDH was exfoliated in the polymer matrix. At higher LDH loadings intercalation of polymer chains into the LDH occurred. The exfoliation of the particles and strong interaction between LDH and PU enabled efficient stress transfer from the PU, which helped improve the mechanical properties. The large aspect ratio of the LDH particles also helped with elastic deformation. These factors increased the static adhesion strength and interfacial stiffness and improved the mechanical properties compared to neat PU. The well-dispersed LDH platelets caused small voids to form during strain. This void formation dissipated energy, which increased the amount of strain the composite could withstand and increase the mechanical properties. At higher LDH loadings, agglomeration decreased the mechanical properties. TGA data showed a reduction in thermal stability, possibly due to the Co-Al LDH catalyzing the degradation of the amide group in PU. However, the barrier effect of the LDH slowed the rate of thermo-oxidised degradation.

Kotal et al. [77] further investigated the effect of stearate-modified LDH (St-LDH) on the properties of PU. They used in situ polymerisation to incorporate the St-LDH into the PU and then crosslinked the mixture. The PU chains were intercalated into the St-LDH, and the LDH layers were exfoliated and well dispersed, although agglomerates started to form at higher loadings. FTIR showed that hydrogen bonding occurred between the LDH and PU and that a urethane linkage formed between the -OH groups of LDH and the -NCO groups of the prepolymer. TS and EB were both increased by the addition of St-LDH. The increase in TS, EB and tensile modulus all reached a maximum at 3 wt % St-LDH before decreasing again due to agglomeration at higher loadings. The increase in mechanical properties occurred at a higher loading of St-LDH than in the previous study [73]. The high surface area of the St-LDH particles, caused in part by exfoliation and hydrogen bonding, led to better polymer–filler interaction, which in turn improved the stress transfer efficiency from the PU and increased TS. The long hydrocarbon chain of the stearate anion had a plasticising effect, which increased EB. Chain slippage and platelet orientation may also have contributed. The tensile modulus was most likely improved due to the large aspect ratio and reinforcing effect of the LDH as well as the formation of shear zones during deformation. The strong interaction between St-LDH and PU also increased the storage modulus and lowered the tan(δ) peak due to the polymer chain movement becoming restricted. At higher LDH loadings, the filler–filler interaction started to dominate over the filler–polymer interaction which decreased the storage modulus. The restricted polymer chain movement also increased T${}_{g}$, but at higher loadings of LDH T${}_{g}$ decreased again due to the plasticising effect of the stearate anion dominating over the reinforcing effect. The barrier effect of the LDH particles also increased the thermal stability of the composites. This study shows that proper dispersion of the LDH in the rubber matrix plays an important role in how much the properties of the PU are improved and that stearate has a plasticising effect in PU.

PU can be used as a shape memory polymer that can maintain a temporary shape after deformation and be restored to its original shape after being exposed to a stimulus such as heat, water or light [78]. The shape memory ability is related to the crystallinity of the soft segments in PU [78]. Phua et al. [79] used two sizes of polydopamine coated Mg-Al LDH (D-LDH), one large and one small, to enhance the mechanical and shape memory properties of polycaprolactone-based PU. The polydopamine coating improved the dispersion of LDH in the PU but agglomeration still occurred at higher concentrations of LDH. Uncoated LDH did not have any beneficial effects on the properties of PU. The D-LDH particles acted as nucleating agents due to the polydopamine coating forming hydrogen bonds with the PU hard segments. The large LDH particles negatively affected strain recovery because they connected hard segments to each other by passing through soft segments. Unlike uncoated LDH, the D-LDH promoted crystallisation on the soft and hard segments of PU, though the smaller LDH particles performed better. The enhanced crystallisation led to better shape memory properties because the stretched soft segment chains had restricted movement, preventing them from relaxing. The hard segment crystallisation and reinforcing effect of the LDH also enhanced stress recovery. The better interaction between the small LDH particles and PU led to more effective stress transfer and therefore better reinforcement. The presence of the coating enhanced the shape memory properties of PU. Particle size and LDH coating both affect the final properties of the composite.

Yu et al. [80] investigated the effect of dodecyl sulphate-modified Mg-Al LDH (DS-LDH) and the presence of dangling chains on PU in a PU/PMMA blend. They found that increasing the LDH content increased the stiffness of the polymer. Chain mobility became more restricted as the free volume in the polymer was reduced by interaction between the LDH and polymer, increasing T${}_{g}$. The damping ability of the polymer blend was increased due to a synergistic effect between the dangling chains of the PU and the LDH. The dangling chains moved with the polymer backbone chain and increased resistance to motion and internal friction between the polymer, dangling chains and filler. The LDH itself also increased internal friction, increasing the damping ability. For this reason the composites with dangling chains had higher tan(δ) values than the composites without dangling chains.

One of the methods that can be used to incorporate LDH into an elastomer such as PU is by using in situ polymerisation. Dispersion of the LDH elastomer affects the mechanical properties, as has been shown in many of the studies already discussed here. Carmo et al. [81] used different methods of dispersing LDH into a PU prepolymer and investigated which method resulted in the best dispersion of the LDH. They used four dispersion sequences:

- TBT: 30 min ultraturrax, 2 h ultrasound bath, 5 min ultraturrax;
- TST: 30 min ultraturrax, 1 min sonication, 5 min ultraturrax;
- T: 30 min ultraturrax;
- B: 2 h ultrasound bath.

They found that the TBT, TST and T methods all promoted exfoliation and intercalation during polymerisation, and that the TBT method was best at promoting this. TBT and T resulted in the least filler–filler network formation, which lowered the Payne effect in these samples. This showed that these methods dispersed the LDH well and promoted polymer–filler interaction. All methods increased the T${}_{g}$ due to the polymer–filler interaction, with the T method increasing it the most. The LDH increased the storage modulus regardless of the dispersion method used. The storage modulus was lowest when the T method was used. This may have been due to the LDH hydroxyl groups interacting with the isocyanate groups and preventing them from participating in the polymerisation, ultimately reducing crosslink density. TBT reduced the amount of free isocyanate the most, meaning it caused the most intercalation into the LDH. The B method did not perform well in comparison to the other methods. Overall, the TBT method was most effective as it could destroy clusters and deagglomerate smaller particles.

Zhang et al. [82] grafted γ-aminopropyltriethoxysilane (APS) onto dodecyl sulphate-modified Mg-Al LDH (APS-DS-LDH) and added it to PU. Grafting APS onto DS-LDH improved the interfacial interaction between the filler and rubber. As the APS-DS-LDH content increased, the T${}_{g}$ of the soft segments decreased. The authors stated that this was due to stronger interaction between the PU and APS-DS-LDH, contrary to what most other studies have observed. The general trend is that the T${}_{g}$ is increased as interaction between the polymer and LDH is increased, restricting chain movement. This observation may be due to interaction between the modifier and polymer, although typically using dodecyl sulphate as a modifier does not decrease the T${}_{g}$. The strong interaction between the filler and polymer increased phase separation between the hard and soft segments of the PU. The damping properties of PU were increased because the internal friction between the polymer and filler increased with increasing APS-DS-LDH content. At higher levels the damping properties decreased again due to agglomeration causing filler–filler interaction to dominate over filler–polymer interaction and reducing the friction between APS-DS-LDH and PU. Hydrogen bonding occurred between the polar groups of the modified LDH and PU increased the interfacial interaction between the polymer and filler. This increased the TS. The strong interfacial interaction between APS-DS-LDH and PU combined with the plasticising effect of the dodecyl sulphate chain increased EB. The APS and dodecyl sulphate hydrocarbon chains increased the flexibility of the PU matrix which caused a decrease in the modulus. APS-DS-LDH also increased the thermal stability of PU.

Work had previously been done on the synergistic effect of carbon nanotubes (CNT) and LDH in silicon rubber [83]. CNT and LDH both tend to form agglomerates in organophilic materials such as rubber [84], limiting their usefulness. Roy et al. [85] investigated the effect of combining Mg-Al LDH with organically modified CNT in a 50:50 wt % ratio PU/NBR blend. They found that at a low loading of 0.5 wt % filler, EB increased by 180% and TS improved by 171%. They modified CNT with sodium dodecyl sulphate and then mixed it with LDH to form a DS-CNT-LDH hybrid. The hybrid filler formed a three-dimensional network and the modified CNTs were uniformly distributed on the surface of the LDH platelets due to the interaction between the LDH and sulphate groups on the modified CNT. The hybrid filler was partially exfoliated in the rubber matrix and a filler network formed in the rubber. At higher filler concentrations, agglomeration started to occur. This decreased the mechanical properties by creating sites for failure initiation. The hybrid filler increased the storage modulus, TS and EB compared to the neat blend. This indicates that DS-CNT-LDH has a reinforcing effect caused by good dispersion and interaction between the rubber and filler that allows efficient stress transfer. The reinforcing effect was more pronounced at lower loadings due to less agglomeration than at higher loadings of DS-CNT-LDH. The polymer–filler interaction also restricted polymer chain mobility which caused an increase in T${}_{g}$ and melting temperature (T${}_{m}$). The crystallisation temperature (T${}_{c}$) was increased due to the DS-CNT-LDH acting as a nucleating agent. The LDH and CNT, when tested individually, improved the mechanical properties of the rubber much less than when the two components were used together. This shows that the LDH and CNT had a syngergistic effect in the rubber.

Roy et al. [86] also created hybrids with Zn-Al LDH and CNT and carbon nanofibers (CNF) by modifying the CNT and CNF with sodium dodecyl sulphate and then mixing them with the LDH. These were added to a 50:50 weight ratio PU/NBR blend. Both the CNT-LDH and CNF-LDH fillers formed uniformly distributed network structures at low loadings, but agglomeration occurred at higher loadings. Both hybrid fillers had a reinforcing effect in the rubber, improving TS and EB. A syngergistic effect between the LDH and modified CNT as well as between the LDH and CNF was observed because the individual components of the hybrids did not improve the mechanical properties as much as the hybrids did. CNT-LDH caused greater improvement in the mechanical properties than CNF-LDH and also increased the T${}_{g}$ more, which shows that it has better interaction with the polymer than CNF-LDH.

Roy et al. [87] did another study using a 50:50 weight ratio PU/NBR blend in which they made hybrids of dodecyl sulphate-modified CNF and Mg-Al LDH. The LDH particles had a high affinity towards the modified CNF and attached to the side walls of the CNF. The DS-CNF-LDH was partially exfoliated in the rubber matrix. There was strong interaction between DS-CNF-LDH and the rubber, which was reflected in the increase in storage modulus, loss modulus and T${}_{g}$, and the decrease in the tan(δ) peak at lower filler concentrations. The strong interfacial interaction also increased TS and EB. The improvement in mechanical properties was higher for the hybrid than when the individual components were used. This is similar to what was observed previously [85,86].

Srivastava et al. [88] compared the ability of MMT and Mg-Al LDH to improve the shape memory behaviour of PU. The MMT was better dispersed than the LDH. Both fillers had a nucleating effect in the soft segments of the PU, which caused crystallisation behaviour, although the MMT performed better. The good dispersion and better crystallisation when MMT was used caused the shape recovery efficiency of the MMT composite to be better. LDH increased the modulus of the PU more than MMT and decreased the TS but slightly less than MMT. The LDH decreased EB the most.

Multiple methods have been used so far to incorporate LDH into PU. Typically the LDH is organically modified and then mixed into the polymer via physical dispersion, solution blending [72,76] or in situ polymerisation [77,82]. In the case of solution blending, the LDH is synthesised, modified and then dispersed in tetrahydrofuran (THF). The PU is then added and the mixture is dried [71]. In the case of in situ polymerisation, the LDH is synthesised and modified and then added to the prepolymer dissolved in THF and then crosslinked [77]. Starukh et al. [89] tried to simplify the process of incorporating LDH into PU by physically modifying the prepolymer linear macro diisocyanate (MDI). Mg-Al LDH was modified with dodecyl sulphate (DS-LDH) and dispersed in MDI diluted in THF. After this, the PU films were prepared using this MDI mixture. The LDH was partially exfoliated and hydrogen bonds most likely formed between MDI and LDH. The TS and EB were both significantly increased by adding the DS-LDH due to the interaction between the polar groups of the LDH and PU. The results were compared to the results of studies conducted with LDH incorporated using in situ polymerisation and physical dispersion and it was found that these results were comparable to when the other methods were used. A direct comparison with the same LDH loading, LDH type and modifier was not performed but this study showed that this method of incorporating LDH into PU is viable.

Table 4 summarises the effect of LDH on the properties of PU.

### 5.5. Natural Rubber (NR)

NR is usually tapped from the rubber tree, *Hevea brasiliensis*, and makes up a significant portion of most tires, especially in high performance tires such as aeroplane tires [90]. It can be crosslinked using sulphur vulcanisation or peroxide cure [91]. It is typically reinforced using carbon black, although silica is also used [92].

Three articles relevant to the literature review criteria have been published, one in 2010, one in 2013 and one in 2021. Zn-Al LDH was used in the first study and Mg-Al LDH was used in the other two. The modifiers used were dodecyl sulphate and hydrogenated fatty acid and in the third study IL was used together with LDH but the LDH was kept unmodified.

Adhha Abdullah et al. [93] compared the effect of Zn-Al LDH modified with dodecyl sulphate (DS-Zn-Al LDH) to unmodified Zn-Al LDH on the properties of NR. They did not compare the results to NR containing no LDH. The NR chains did not intercalate into the unmodified LDH and the LDH was present as agglomerates in the rubber matrix. Organic modification increased the interlayer spacing of the LDH, which allowed NR chains to intercalate. The DS-Zn-Al LDH was also exfoliated in the NR. In the case of unmodified LDH, the TS decreased with increasing LDH content. The DS-Zn-Al LDH was better dispersed and intercalated, and had better interfacial interaction with the NR, which caused an increase in TS. The TS of the modified LDH composites was higher than for the DS-Zn-Al LDH composites at the same loading. Increasing the DS-Zn-Al LDH content improved the TS up to a maximum increase at 7 phr before decreasing again. The decrease in TS at higher loadings was most likely caused by the formation of agglomerates or poor dispersion. This work showed that organic modification improved the reinforcing properties of LDH due to increased compatibility, and created the possibility of exfoliation and intercalation of polymer chains into the LDH.

MMT is also a layered clay but is cationic, whereas LDH is anionic. Modified and unmodified MMT and Mg-Al LDH were added to a NR/cis-1,4-polybutadiene blend to determine the effect of the clays on the mechanical properties and curing behaviour of the rubber by Bottazzo et al. [94]. They modified the LDH with a hydrogenated fatty acid (HFA-LDH) and the MMT with dimethyl dihydrogenated tallow quaternary ammonium and methyl tallow bis-2-hydroxyethyl quaternary ammonium. The organic modification of both clay types improved the interaction between the clay and rubber and increased crosslinking. Both modified and unmodified LDH increased the scorch time and CRI. This may be due to the LDH inactivating the vulcanisation agent partially, increasing the scorch time, and then acting as a catalyst and increasing the CRI. This behaviour may be due to the formation of coordination complexes between the LDH and ZnO, as discussed by Thakur et al. [57]. The anionic modifier on the LDH slowed the curing reaction, whereas the organic modifiers on MMT accelerated it. This could be seen in the lower CRI of the modified LDH composites. Both the modified and unmodified LDH decreased the TS of the rubber while the organically modified MMT increased the TS. The poor TS of the LDH composites may be due to the formation of agglomerates and poor interaction with the rubber. All the clays decreased EB because the clays themselves and the increased crosslink density increased the stiffness of the rubber. The organically modified LDH also reduced abrasion resistance due to poor dispersion in the polymer matrix. These results show that both the nature of the clay and the organic modifier play a role in how the properties of the rubber are affected.

The same group that worked on using ILs in XNBR cured with LDH also compared the effect of ILs on the properties of NR composites containing Mg-Al LDH, cellulose and silica [95]. The focus of this discussion will be on the composites containing LDH. They compared 1-butyl-3-methylimidazolium bromide (BmiBr) and 1-butyl-1-methylpyrrolidinium bromide (BmpyrBr) at the same concentrations. FTIR showed that the LDH formed filler–filler and polymer–filler interactions and that the addition of the ILs did not affect these interactions. In comparison to neat NR, adding LDH lowered the t${}_{90}$ and adding BmiBr to the LDH composite further decreased it, whereas adding BmpyrBr increased the t${}_{90}$ to a value similar to neat NBR. The scorch time was lowered slightly in the LDH composites compared to neat NR and both ILs decreased it further to a small extent. The maximum torque was increased by adding ILs which may have been due to an increase in crosslink density. In comparison to when silica was used as filler, the effect of the LDH was less pronounced. The cellulose results were similar to that of the LDH composites. Swelling tests showed that, compared to neat NR, LDH caused a significant increase in crosslink density. This may have been due to the LDH participating in the crosslinking by acting as an activator, similarly to ZnO, by providing metal ions. The authors suggested that the alkaline nature of LDH may also have played a role, since sulphur vulcanisation favours alkaline conditions. The other two fillers are both hydrophilic and as such were not compatible with the NR and did not increase the crosslink density as much. Adding the ILs further increased the crosslink density regardless of filler type. BmiBr increased the crosslink densities more than BmpyrBr did. The increase in crosslink density when ILs were added was due to the improved dispersion of the crosslinking additives in the rubber matrix, improving the efficiency of the crosslinking process. The T${}_{g}$ was not affected by the three fillers or by the ILs. The authors reported that the differences in T${}_{g}$ were within the range of experimental error and as such the influence of these additives was not significant. Adding LDH to the NR increased the modulus and TS and decreased the EB. The ILs, in combination with LDH, further increased the modulus and TS, with BmiBr increasing it the most. The ILs also further decreased the EB, with BmiBr decreasing it the most. This corresponds to the changes in crosslink density of the composites containing LDH. In comparison to the other fillers, LDH had the greatest reinforcing effect as well as the highest crosslink density, which resulted in these composites having the highest TSs. The EB of the filled composites was decreased due to the increased stiffness caused by the presence of the fillers and the increased crosslink density. The fillers decreased the tan(δ) peak due to the increased stiffness that reduced chain mobility. LDH influenced the tan(δ) peak the least and silica the most. The ILs did not affect the tan(δ) values. Addition of ILs decreased the thermal stability of all the composites, with BmpyrBr decreasing it the most. Silica and cellulose increased the onset decomposition temperature whereas LDH decreased it. The NR composites remained thermally stable up to 300 ${}^{\circ }$C regardless of filler type. This work showed that ILs can enhance the mechanical properties of NR composites containing these three fillers and help to increase the stiffness of the rubber composites.

Table 5 summarises the effect of LDH on the properties of NR. In the first entry, the only comparison available was between modified and unmodified LDH and the percentage change is therefore from unmodified LDH to modified LDH rather than from neat NR to NR containing LDH like the other entries.

### 5.6. Silicone Rubber (SR)

SR contains polar Si-O groups in the backbone, making it unique in having both organic and inorganic properties. SR can be crosslinked using organic and metal peroxide crosslinking or via electron beam crosslinking [47,96]. The Si-O groups in this rubber give it very good heat, cold, abrasion and ozone resistance, chemical stability, weatherability and electrical insulation properties, making SRs useful in many fields, such as the automotive, construction, electronic and food industries [97].

There are four articles on the effect of LDH on the mechanical properties of SR. They were published in 2011, 2012, 2014 and 2020. The LDHs used in SR were Mg-Al LDH, Li-Al LDH and Co-Al LDH, and the modifiers used were dodecyl sulphate and stearate, although unmodified LDHs were also used.

Inorganic nanofillers such as LDH can be used to improve the properties of SR. Pradhan et al. [98] modified Mg-Al LDH with dodecyl sulphate (DS-LDH) and found that compared to neat SR, addition of DS-LDH improved the TS and EB, toughness, thermal stability and solvent uptake resistance, although the properties decreased at higher loadings due to agglomeration of the DS-LDH particles. The DS-LDH particles were exfoliated in the SR, which may have been in part due to hydrogen bonding between the LDH hydroxyl groups and the SR. At low loadings the DS-LDH was well dispersed. The increase in mechanical properties was due to the interactions between the rubber and LDH as well as the exfoliation and good dispersion of the LDH. The platelet orientation and chain slippage of DS-LDH in the rubber matrix helped increase the EB. The particles improved the mechanical properties because they could absorb energy during deformation and interrupted crack propagation. The strong interaction between the DS-LDH and SR could also be seen in the increase in T${}_{m}$ at low LDH loadings. Agglomeration decreased the T${}_{m}$ at higher LDH loadings. The crystallinity of the SR was increased due to the DS-LDH particles acting as nucleating agents, though agglomeration at higher DS-LDH loadings decreased the crystallinity.

Pradhan et al. [99] also added stearate-modified Mg-Al LDH (St-LDH) to SR. At low concentrations the LDH was uniformly dispersed and exfoliated. At higher loadings agglomeration started to occur. There was also evidence of hydrogen bonding between St-LDH and SR. The thermal stability of SR was increased by adding St-LDH because the exfoliated layers of LDH acted as a barrier to volatile degradation products. As the LDH loading was increased the thermal stability decreased. This was most likely due to the formation of agglomerates and decreased exfoliation, as well as the decomposition of the organic modifier and the particles acting as heat source domains. St-LDH had a nucleating effect in SR and the T${}_{c}$ increased with increasing LDH content. The T${}_{m}$ increased, reached a maximum and then decreased with increasing St-LDH content. The increase in T${}_{m}$ may have been due to the interaction between the hydroxyl groups of the LDH and the SR restricting chain movement. TS, modulus and EB of SR were also increased by the St-LDH, with a maximum increase at 3 wt %. Agglomeration reduced the mechanical properties at higher loadings of St-LDH. The TS and storage modulus were increased by the large aspect ratio of St-LDH particles and the polymer–filler interaction. Chain slippage and platelet orientation increased the EB. The stearate modifier had a plasticising effect in the SR and lowered the T${}_{g}$.

Organically modified LDH has been successfully incorporated into SR. Another filler of interest in rubber is CNT [84]. Pradhan and Srivastava [83] prepared multiwalled carbon nanotube (MWCNT) hybrids with unmodified Li-Al LDH, Mg-Al LDH and Co-Al LDH by dry grinding them together. The Li-LDH-MWCNT was not homogeneously dispersed in SR but Mg-LDH-MWCNT and Co-LDH-MWCNT were better dispersed. Mg-LDH-MWCNT was dispersed best. The LDH/MWCNT hybrids formed a three-dimensional network inside the SR. Mg-LDH-MWCNT had the largest surface area and Li-LDH-MWCNT had the smallest surface area. The larger surface area could have assisted in dispersing the MWCNT in the SR. The hybrid fillers all increased the TS and EB of the SR and the improvement reached a maximum before decreasing again due to agglomeration. Mg-LDH-MWCNT was well dispersed and had a large surface area, which resulted in strong interaction and good stress transfer between it and the rubber. This caused the Mg-LDH-MWCNT to increase the TS more than the other fillers. The increase in EB was due to the entanglement of SR chains, chain slippage, platelet orientation and the deformation of MWCNT. In comparison to neat LDH and neat MWCNT, the improvement in mechanical properties was better when the hybrids were used. This showed that LDH and the MWCNT had a synergistic effect in SR. Surface fracture morphology showed that Mg-LDH-MWCNT had the strongest interaction with SR and Li-LDH-MWCNT had the weakest interaction since the Li-LDH-MWCNT particles were pulled out of the rubber matrix and Mg-LDH-MWCNT particles were not. The hybrid fillers also increased T${}_{g}$ and T${}_{m}$ by restricting the movement of polymer chains. T${}_{c}$ was increased as well, which indicated that the hybrid fillers acted as nucleating agents. The neat LDHs and MWCNT did not have the same effect on T${}_{g}$, T${}_{m}$ and T${}_{c}$, which again shows the synergy between the LDH and MWCNT. Mg-LDH-MWCNT increased the thermal stability and crosslink density of SR the most due to its good dispersion and interaction with SR. The synergy between LDH and MWCNT can prove useful in the reinforcement of rubber.

Anti-tracking agents are added to SR to reduce tracking and erosion failure but are incompatible with SR and require high loadings to be effective, thus reducing the mechanical properties of SR. In their previous work, Wu et al. [100] found that silane-containing additives improved tracking and erosion resistance of SR [101,102] so they added vinyltriethoxysilane (ViTES) and Mg-Al LDH to high temperature vulcanised SR and found that ViTES and LDH have a synergistic effect in SR. The ViTES ethoxy groups hydrolysed into hydroxyl groups and condensed with the LDH hydroxyl groups, forming chemical bonds. This improved dispersion of the LDH. The ViTES acted as a bridge between the LDH and SR and participated in the crosslinking reaction. When both ViTES and LDH were added to SR the TS, EB and tear strength were improved but when they were added separately the properties deteriorated. This showed that the combination improved interfacial bonding with SR and thus improved stress transfer. Individually, ViTES and LDH did not improve tracking, but together they improved tracking and erosion resistance.

Table 6 summarises the effect of various types of LDH and modifiers on the properties of SR.

### 5.7. Nitrile Butadiene Rubber (NBR)

NBR is an acrylonitrile and butadiene copolymer, and as such has double bonds in the backbone chain. Due to these unsaturated bonds, NBR can undergo sulphur vulcanisation [103] and peroxide vulcanisation [104]. It is resistant to oil absorption, but oxidises very easily, so aging resistance can be a concern [3].

**Figure 10:**
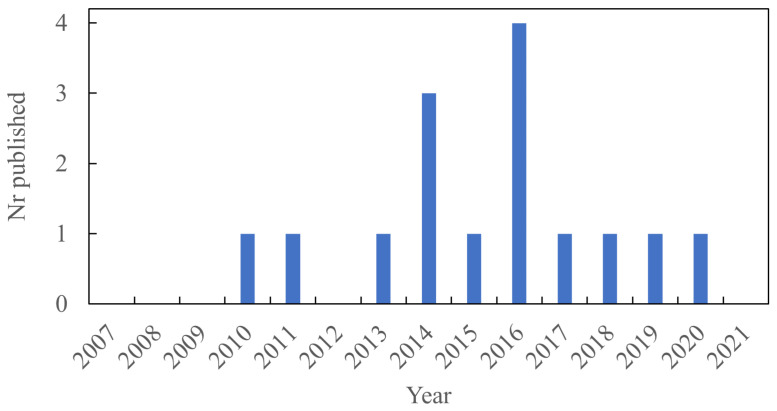
Number of papers published on LDH in NBR per year.

There are 15 studies in which NBR was used, as shown in Figure 10, although four of these articles were on PU/NBR blends and were discussed in the section on PU.

The first study that used NBR was conducted by Kotal et al. [41] where Mg-Al LDH modified with dodecyl sulphate was used in a 50:50 wt % PU/NBR blend, as discussed in the PU section.

While stearic acid can be used to modify LDH simply to increase the interlayer spacing and compatibilise the LDH with the rubber matrix, it is also a component of sulphur cure systems. Another important component of the sulphur cure system is ZnO. In recent years, there has been growing concern over the effect that ZnO has on aquatic life and an effort has been made to reduce the levels of ZnO in rubber [105,106,107]. Das et al. [16] synthesised Mg-Zn-Al LDH modified with stearic acid and added it to NBR to determine whether it can replace the stearic acid and ZnO used in the sulphur cure system. Other elastomers, SSBR, CR, NR and XNBR, were also tested but the focus of the article was on NBR. They compared the ZnO and stearic acid free composites to a control sample with stearic acid and ZnO as well as one that also contained carbon black. They found that partial intercalation and exfoliation took place in the NBR. Due to this exfoliation, the LDH had a large surface area so more of the Zn ions were available to participate in the crosslinking process. In comparison, most of the ZnO ions are in the bulk crystal and cannot participate in the crosslinking reaction. This means less Zn is needed when the LDH is used instead of ZnO to obtain the same results. The LDH cure was also more efficient than the conventional cure system despite having ten times less Zn than the conventional cure system. The TS of the LDH-cured composite was comparable to the ZnO and stearic acid-cured composite, but the EB was lower. This was most likely due to the increase in crosslink density of the LDH-cured composite. When NBR containing carbon black was cured with the LDH, the mechanical properties were not significantly affected. The LDH-cured composite also retained its properties better after aging than the conventionally cured NBR. This may be due to the LDH layers acting as a barrier to oxygen, slowing down oxidative cleavage. Interestingly, the LDH composite was transparent. This shows that LDH has the potential to act as a functional filler that can be used to reduce the amount of ZnO used to cure rubber.

Feng et al. [108] investigated the effect of two different organically modified LDHs, styrene sulphonate-modified LDH (SS-LDH) and dodecyl benzenesulphonate-modified LDH (DBS-LDH), on the properties of NBR and compared it to unmodified LDH. Some polymer chain intercalation occurred in SS-LDH but not in DBS-LDH, which in fact saw a decrease in basal spacing and was poorly dispersed. Unmodified LDH also did not have any polymer chains intercalated due to the small interlayer spacing and the poor interaction between the LDH and the rubber. This also caused agglomeration and poor dispersion of the LDH. The NBR was peroxide-cured. The SS-LDH delayed the curing process which was most likely due to the fact that the modifier had a double bond that participated in the curing reaction. This caused the crosslink density of the SS-LDH composite to be higher than the other composites despite the slower cure and was also the reason that SS-LDH had better interaction with NBR than the other LDHs. DBS-LDH and LDH decreased the cure times compared to neat NBR. The basic nature of the LDH could be favourable to the homolytic cleavage of peroxide, which could then accelerate the cure. The good interaction between SS-LDH and NBR led to this composite having the highest TS. The EB was slightly decreased but not significantly. Both organic modifiers caused an increase in T${}_{g}$ due to improved interaction with the rubber. Unmodified LDH had a very small increase in T${}_{g}$. This study shows that the nature of the organic modifier plays a role in how effective the LDH is as a reinforcing filler in NBR.

The effect of using the surfactant Pluronic F-127 on the dispersion of Mg-Al LDH in NBR latex was investigated by Braga et al. [109]. The LDH adsorbed the hydrophilic groups of the surfactant through hydrogen bonding and the hydrophobic groups of the surfactant then acted as a barrier between LDH particles. This improved their dispersion in NBR and reduced agglomeration in comparison to when no surfactant was used. NBR chains were also intercalated into the LDH layers.

The same research group that used lignin-modified LDH in SBR [110] also investigated the effect of using lignosulphonate, a byproduct of the pulping industry, as a modifier for Mg-Al LDH in NBR [111]. Compared to neat LDH, the lignosulphonate-modified LDH (LS-LDH) was better dispersed in NBR and did not form agglomerates. This translated to a larger increase in TS and tear strength, although EB was lower than for the unmodified LDH composite. Contrary to what has been observed in most other studies, the T${}_{g}$ was decreased by unmodified LDH as well as LS-LDH, although the LS-LDH decreased the T${}_{g}$ less, showing that LS-LDH had better interaction with NBR.

Previous work showed that LDH containing Zn could be used to replace ZnO in rubber [16]. Stearate-modified Zn-Al LDH was used as a replacement for ZnO in NBR in a sulphur cure and a peroxide cure system by Eshwaran et al. [112]. The acrylonitrile (ACN) content of the NBR was also varied. The LDH was partially exfoliated in the rubber and some of the rubber chains intercalated into the LDH. Despite having a lower concentration of Zn ions in the LDH sulphur cure system than the conventional system with ZnO, the cure was still efficient. At low ACN content, the LDH sulphur cure system performed better than conventional sulphur and peroxide cures. At high ACN content, and therefore high polarity, the LDH peroxide cure was the most efficient. In general, however, the optimum cure time for the peroxide and the curing reactions were not affected by the LDH. The NBR composites containing LDH had higher moduli and TS values at all ACN content levels than the conventionally cured composites while the EB values were lower. After aging, the LDH-cured composites also retained their mechanical properties better. This may be due to the LDH acting as a barrier to oxygen diffusion which slowed down degradation. This shows that ZnO can be replaced by Zn-Al LDH in a sulphur cure system and that Zn-Al LDH can also be added to peroxide cured composites without negatively impacting the cure system and can, in fact, be used to improve the properties of the composites.

Eshwaran et al. [113] investigated the effect of stearate modification of Zn-Mg-Al LDH (St-LDH) on the properties of NBR where unmodified LDH and St-LDH was used to replace ZnO and stearic acid in the curing system. A commercial unmodified LDH, a commercial stearate-modified Zn-Al LDH and a lab synthesised stearate-modified Zn-LDH were used and compared to a stearic acid and ZnO cure system with Zn levels equivalent to that of the LDHs. Despite there being no stearic acid present when the unmodified LDH was used, the NBR still cured but the scorch time was decreased due to the absence of stearate groups. The cure rate of the lab synthesised St-LDH was the highest. The excess stearate anions made the formation of zinc stearate coordination complexes more effective. The crosslink density was reduced by the modified LDHs compared to unmodified LDH but the crosslink densities of these composites were still higher than the ZnO and stearic acid-cured composite. A higher stearate content decreased the crosslink density. Unmodified LDH increased the mechanical properties the most. The stearate groups may have acted as internal lubricants and this effect dominated over the reinforcing effect caused by polymer chain intercalation and the platelets themselves, decreasing the mechanical properties. A larger number of stearate groups caused a greater reduction in mechanical properties. The LDH platelets may also have collapsed as the Zn and stearate groups participated in the curing process, reducing the reinforcing effect of the LDH. The modified LDHs still outperformed the ZnO and stearic acid-cured composite. The modified lab synthesised LDH reduced T${}_{g}$ due to the lubricating effect of the stearate chains. At equivalent levels of Zn, the Zn-AL LDHs performed better as part of the vulcanisation process but the stearate groups negatively impacted the mechanical properties due to their lubricating effect.

Three studies were conducted with 50:50 wt % PU/NBR blends where Mg-Al LDH together with organically modified CNT [85], Zn-Al LDH together with CNT and CNF [86] and Mg-Al LDH with CNF [87] were used, as discussed in the PU section.

He et al. [114] modified Mg-Al LDH with sodium p-styrenesulphonate hydrate (SSS-LDH) and investigated the effect of this LDH on the aging behaviour of NBR. SSS-LDH was compared to unmodified LDH. Unlike in most work performed previously, carbon black was also added to all the formulations. The composites with unmodified LDH had higher TS than the composites containing SSS-LDH. In both cases TS decreased with increasing LDH content. The unmodified LDH still improved the TS compared to the LDH free composite but the SSS-LDH decreased the TS to values below that of the LDH free composite. The authors mention that the unmodified LDH may have acted as an accelerator in the sulphur vulcanisation while the SSS-LDH acted as a suppressant. A similar trend was observed for the EB. After aging, however, the SSS-LDH composite had the highest TS and EB. This may be because the vulcanisation process continued during aging. The additional interactions between SSS-LDH and NBR then increased the strength of the interfacial interaction. The saturation of NBR was also increased, which reduced chain scission during aging. The accelerating effect of unmodified LDH, however, caused the vulcanisation to reach saturation before aging and could have caused over-vulcanisation. There was also little interaction between the LDH and NBR, which allowed microvoids to form around the LDH particles. This could have allowed more oxygen to penetrate into the rubber and cause a reduction in mechanical properties.

Wang et al. [115] used Mg-Al LDH surface modified with polyvinylpyrrolidone (PVP) to improve the gas barrier properties of NBR and used layer-by-layer assembly to incorporate the LDH into the NBR. LDHs have good gas barrier properties due to the large aspect ratios of the platelets. They found that the oxygen permeability was decreased by the LDH and that the LDH improved the thermal stability and TS compared to neat NBR and slightly reduced EB.

LDHs have proven to be useful in improving the mechanical properties of NBR and can also be used to improve the aging resistance. Li et al. [116] modified Mg-Al LDH with 4 amino-benzenesulphonic acid monosodium salt (SAS-LDH) and used it to improve the aging resistance of NBR. During mixing, and even after aging, some NBR chains were intercalated into the SAS-LDH and the SAS-LDH was exfoliated. This did not happen when unmodified LDH was used. Both LDHs increased EB and TS compared to neat NBR. Before aging the unmodified LDH composite had better mechanical properties because the LDH accelerated the vulcanisation process. However, in comparison to unmodified LDH and unfilled NBR, the SAS-LDH composite had a smaller decrease in TS and EB after aging and therefore had the best mechanical properties after aging. The unmodified LDH composite may have aged faster due to the poor interaction between the LDH and polymer causing microvoids to form around the LDH particles, allowing more oxygen to penetrate into the NBR. The microvoids also decreased the mechanical properties. The modified NBR composites did not form any microvoids due to better interaction between the LDH and NBR. SAS-LDH also increased the thermal stability, T${}_{g}$ and storage modulus due to the strong interaction between NBR and SAS-LDH.

The dispersion of LDH and the interaction between the LDH and rubber both affect the mechanical properties of the composites. Maciejewska and Sowińska [117] used ILs (BMpyrrolBF4, BMpyrBF4 and BMpipBF4) to improve the dispersion of Mg-Al LDH in NBR. They compared the composites containing LDH and IL with one containing carbon black and one containing silica. The LDH composite had the lowest thermal stability compared to when carbon black or silica was used as filler because LDH starts decomposing above 100 ${}^{\circ }$C. Adding ILs decreased the thermal stability for all three composite types. NBR containing LDH had the lowest onset temperature of vulcanisation. The authors stated that this may be because LDH is alkaline and sulphur vulcanisation prefers alkaline conditions. The nature of the IL also determined how the LDH affected vulcanisation. BMpyrrolBF4 and BMpipBF4 did not affect the onset temperature but did reduce the enthalpy of vulcanisation. BMpyrBF4 increased the vulcanisation temperature. All three ILs lowered the temperature at which the post-curing reactions took place. BMpyrBF4 also made the crosslink distribution in the LDH composite more homogeneous but did not affect the crosslink density. This may be because the BMpyrBF4 improved the dispersion of LDH and crosslinking agents in the rubber matrix. The effect of LDH on the storage modulus was not changed by the ILs. All three filler types increased the storage modulus, with silica increasing it the most. Carbon black and LDH increased the T${}_{g}$ similarly and the ILs did not affect the T${}_{g}$ significantly.

Previous work had shown that modifying LDH with SSS or SAS improved the aging resistance of NBR [114,116]. The same research group then also focused on synthesising a multi-modified LDH to improve the aging resistance of NBR [118]. 4-Aminodiphenylamine (ADPA) is a chemical intermediate for anti-aging compounds that can be grafted onto hydroxy-rich surface carriers such as graphene oxide by using a silane coupling agent [119]. LDH was grown on graphene oxide (GO) and ADPA was reacted with a silane coupling agent, KH560, and grafted onto the GO-LDH. The modified LDH improved the modulus, TS and EB. After aging, the composites containing LDH retained their mechanical properties better than the composites without LDH. This indicates that the modified LDH slowed down the aging process. Adding the modified LDH to composites containing carbon black did not negatively affect the reinforcing properties of carbon black. The strong interaction between the modified LDH and NBR caused physical crosslinks to form, increasing the crosslink density of NBR. Further crosslinking during aging was slowed down by the ADPA on the modified LDH. A modified LDH can therefore be used as an anti-aging compound in NBR.

The effect of different types of LDH on the mechanical properties of NBR are summarised in Table 7. The four studies in which PU/NBR blends were used were summarised in Table 4 and are repeated here. The study by Braga et al. [109] is not summarised here as mechanical property data were not reported.

### 5.8. Hydrogenated Nitrile Butadiene Rubber (HNBR)

HNBR is made by removing double bonds in the backbone chain of NBR, which makes it more resistant to oxidation than NBR and improves the tensile and tear strength [120]. It can be crosslinked via sulphur vulcanisation or peroxide cure [121].

According to the search criteria used, two articles have been published on LDH in HNBR, one in 2012 and the other in 2019. In the first study, Mg-Al LDH was used and in the second Zn-Al LDH was used.

Coagents are functional monomers that are added to rubbers such as HNBR that can be peroxide-cured and are used to improve the properties of the rubber [122]. They are highly reactive towards the free radicals formed during peroxide vulcanisation and can participate in the vulcanisation process [123]. MacIejewska et al. [124] mixed the coagent itaconic acid (IA) with either Mg-Al LDH, MgO or CaO to allow these fillers to participate in the crosslinking process. The modified fillers were compared to their unmodified counterparts. The IA modification reduced the tendency of the filler particles to agglomerate, but all three fillers still formed agglomerates in the HNBR and dispersion was not homogeneous. The LDH agglomerated the most. However, especially in the case of the LDH, the IA modification allowed it to interact strongly with the rubber matrix and improve the mechanical properties despite the agglomeration and poor dispersion observed. The vulcanisation time was decreased by all three fillers and the LDH composite had the longest cure time. The long cure time of the LDH composite may be due to the agglomeration reducing the available surface area and causing it to be less active in the vulcanisation process. The modified MgO and LDH increased the crosslink density the most but the unmodified fillers did not affect the crosslink density at all. The increase in crosslink density by the modified fillers could also be seen in an increase in T${}_{g}$. The modified MgO composite had the highest crosslink density and T${}_{g}$. From this it can be concluded that both the filler type and the modification play a role in how the vulcanisation process is affected. The modified LDH improved the TS of the HNBR even at high loadings despite that composite having fewer ionic crosslinks than the modified MgO and CaO composites. This indicates that the LDH also has a reinforcing effect through stress transfer from the HNBR.

Work had previously been done on SiO${}_{2}$/LDH hybrid fillers in SSBR/SBR blends [125,126] and it became clear that combining LDH with SiO${}_{2}$ had a positive effect on the mechanical properties of the composites. Wang et al. [127] created Zn-Al LDH/SiO${}_{2}$ hybrids by growing the LDH platelets on the SiO${}_{2}$ particles and added it to HNBR. The LDH to SiO${}_{2}$ ratio was varied. The LDH platelets were effective in reducing the agglomeration of SiO${}_{2}$ and improving dispersion in the rubber. As the amount of LDH on the SiO${}_{2}$ was increased, the dispersion of the particles improved. This improvement reached a maximum and then decreased again when too many LDH platelets were present and caused stacking. As was found in the previous work [126], the LDH platelets weakened the filler–filler network, which improved the overall properties of the composite. The platelet structure of the LDH also increased the contact between the rubber and filler. There was strong interaction between the LDH and HNBR, and LDH platelets were entangled with polymer chains. This interaction improved the mechanical properties of the rubber through more efficient stress transfer. The strong filler–polymer interaction restricted polymer chain movement and decreased EB. The Zn in the LDH also acted as an accelerator in the vulcanisation process.

The effect of LDH on the mechanical properties of HNBR is summarised in Table 8.

### 5.9. Solution Styrene Butadiene Rubber (SSBR) and Styrene Butadiene Rubber (SBR)

There are two methods of synthesising styrene butadiene rubber. The first is an emulsion process to synthesise SBR, and the second is to use a solution polymerisation process where an alkyllithium catalyst and anionic polymerisation is used to synthesise SSBR [120,128]. These rubbers are non-polar styrene and butadiene copolymers [129]. SBR is the most widely used synthetic rubber in the tire industry. It has good wet skid and traction properties while still having good abrasion resistance [3]. This makes it the popular choice for tire treads. SSBR has been gaining in popularity because the solution polymerisation process is easier to fine-tune and therefore results in a narrower distribution of molecular weight and controlled monomer distribution [129]. The better controlled reaction conditions result in better properties such as thermal and abrasion resistance, and lower hysteresis loss [130,131]. SBR and SSBR can be crosslinked using sulphur vulcanisation [47], peroxide crosslinking or radiation curing [104].

There are five articles that satisfied the criteria of the literature review. The studies were published, one per year, in 2012, 2013, 2017, 2018 and 2019. In one study Zn-Al LDH was used and Mg-Al LDH was used in the rest.

Das et al. [132] created highly filled SSBR composites containing Zn-Al LDH coated in stearic acid and found that the composites underwent thermotropic behaviour. ZnO was replaced by the LDH. The TS, EB and modulus all increased with an increase in LDH content up to 80 phr. The LDH was uniformly dispersed even at 100 phr but was not exfoliated and the polymer chains did not intercalate into the LDH. As the LDH content was increased, the composites became more stiff due to the restricted chain movement. The thermotropic effect was affected by the concentration of LDH in the composite. At 4 phr there was no thermotropic effect but the effect increased until 60 phr where it reached a plateau.

In most work conducted so far, LDH was modified with sodium dodecyl sulphate, sodium stearate or sodium dodecyl benzenesulphonate to compatibilise the LDH with the rubber and allow for intercalation and exfoliation. Xiao et al. [110] modified Mg-Al-LDH with lignin in an attempt to find a cheap method of organically modifying the LDH. The effect of the modified LDH on the properties of SBR was investigated. The CRI and scorch time of the rubber decreased as the lignin loading was increased, showing that the lignin has an effect on the cure characteristics of the rubber. They also found that lignin-modified LDH had a better effect on the mechanical properties of the rubber than unmodified LDH. This is because the lignin compatibilised the LDH with the rubber and improved the dispersion of the LDH in the rubber matrix. The stress transfer between the rubber and LDH was improved by using lignin, thereby increasing mechanical properties such as stiffness. Modifying the LDH with lignin did not affect the T${}_{g}$ of the rubber very much, showing that there was limited interaction between the modified LDH and the rubber. At low lignin concentration the T${}_{g}$ was increased slightly but at higher lignin concentration the T${}_{g}$ was lowered. LDH also had the effect of thermally stabilising the SBR due to the barrier effect of the LDH platelets inhibiting the release of volatile degradation products. The lignin-modifidied LDH increased the thermal stability slightly more than the unmodified LDH, most likely due to the improved dispersion of the LDH. LDH can therefore be modified cheaply and still be used to produce composites with good mechanical properties and thermal stability.

Lv et al. [133] modified the surface of Mg-Al LDH using fluorescein sodium salt (FLU-LDH) and used it to study the nonlinear viscoelastic behaviour and filler structure in SBR. The fluorescent nature of the modifier enabled the authors to visualise the three-dimensional filler network structure under strain. They used two different sizes of LDH, one with nanosheets (F-NLDH) and the other with microsheets (F-MLDH). TEM showed that the F-LDH particles were uniformly dispersed and a particle network structure formed as the concentration of LDH in the SBR was increased to 15 wt %. When the larger F-LDH was used, no network structure formed even at a relatively high loading of 20 wt %. Both types of LDH increased the storage modulus though the F-NLDH had a larger effect on the storage modulus due to it having a larger specific surface area. When the loading of F-NLDH was increased from 10 wt % to 15 wt % there was a jump in storage modulus which indicated that a network structure formed. Unlike when silica or carbon black are used as filler, the storage modulus only increased slightly upon further addition of LDH because it formed a weaker filler structure than these other fillers. Under strain, the storage modulus remained constant until the critical strain value was reached, after which it decreased rapidly with increasing strain. This dependence of storage modulus on strain is known as the Payne effect. At strain greater than the critical strain, the filler structure that formed could be destroyed. At loadings lower than 15 wt % there was no network structure so the Payne effect could rather be explained by molecular disentanglement than the breakdown of the filler structure. The rubber–filler interaction also did not contribute to the Payne effect because the reversing heat capacity step of the rubber in the LDH/SBR composites was very small for all loadings of LDH. Laser scanning confocal microscopy (LSCM) showed that F-NLDH agglomerates were uniformly distributed throughout the rubber matrix. After stretching the composites to 100% strain, it appeared that the F-NLDH became more homogeneously distributed. The agglomerates became smaller, which showed that stretching broke down the filler agglomerates. However, deagglomeration did not play a large role in the non-linear viscoelastic behaviour of the composite. This is because the rheological response did not reflect the breakdown of agglomerates and could be excluded from the Payne effect. Increasing the F-NLDH loading increased the agglomerate size up to 10 wt %, after which the agglomerate sizes remained consistent. The packing structure of the LDH became closer with increasing LDH loading. This again pointed to the formation of a filler network. The larger F-MLDH particles had larger distances between aggregates and smaller aggregates after stretching. The distance between agglomerates decreased with increasing F-MLDH content but the agglomerate size remained mostly constant. After strain the inter-agglomerate distance did not increase very much (only 10%) which showed that there was no sliding between the SBR chains and F-MLDH particles. The weak interaction between the rubber and LDH meant that little force was needed to cause macromolecular disentanglement.

SiO${}_{2}$ has been used as an alternative reinforcing filler to carbon black, but it tends to form agglomerates in rubber [134]. This reduces the mechanical properties of the rubber composite, so improving the dispersion of SiO${}_{2}$ in rubber is desirable. Kong et al. [125] assembled SiO${}_{2}$ nanodots with Mg-Al LDH using sodium dodecylbenzenesulphonate as a dispersant for the LDH and added it to a 3.2:1 weight ratio SSBR/BR blend. The SiO${}_{2}$ nanodots were uniformly distributed on the surface of the LDH platelets and interacted with the hydroxyl groups of the LDH. This restricted the growth of SiO${}_{2}$ particles and promoted even distribution in the rubber matrix. The SiO${}_{2}$-LDH composites had a higher crosslink density and better interaction between the filler and rubber than when only LDH or SiO${}_{2}$ was used. The SiO${}_{2}$-LDH interacted better with the coupling agent (Si69) which decreased the absorption of the accelerator or curing agents during vulcanisation. This in turn decreased the scorch time and optimum curing time. SiO${}_{2}$-LDH can therefore accelerate vulcanisation. The SiO${}_{2}$-LDH composite had the highest TS and a higher EB than when SiO${}_{2}$ was used alone. The stress transfer and filler–rubber interaction was therefore improved by combining the LDH and SiO${}_{2}$. This was also reflected in the increased storage modulus. Comparing LDH, SiO${}_{2}$, SiO${}_{2}$-LDH and neat SSBR/BR, the SiO${}_{2}$-LDH composite had the highest tan(δ) value at 0 ${}^{\circ }$C, which means it had the highest wet skid resistance, and the lowest tan(δ) value at 60 ${}^{\circ }$C, which means it has the lowest rolling resistance. The heat buildup was also less in the SiO${}_{2}$-LDH composite when compared to only SiO${}_{2}$. This study shows that combining SiO${}_{2}$ with LDH can have a beneficial effect on the properties of the rubber.

The same research group continued the work on SiO${}_{2}$ combined with Mg-Al LDH [126]. In this study they grew the LDH platelets in the surface of SiO${}_{2}$ instead of mixing the two components together, and no organic dispersant or modifier was used. The ratio of LDH to SiO${}_{2}$ was also varied. The SiO${}_{2}$-LDH was uniformly distributed in the SSBR/SBR blend. As the LDH content in the hybrid is increased the shear modulus decreases, which shows the LDH helped reduce the formation of a filler–filler network. At a high level of LDH, the SiO${}_{2}$-LDH composite had an even lower shear modulus than when only LDH was used as a filler. This happened because the LDH platelets grew vertically on the SiO${}_{2}$ surfaces, insulating them from each other. Combining the LDH with SiO${}_{2}$ also increased the tensile modulus, TS and EB of the composites when compared to using only SiO${}_{2}$ due to better dispersion. Adding LDH to SiO${}_{2}$ therefore makes the reinforcing effect of the filler more effective.

Table 9 summarises the effect of various LDHs on the properties of SSBR and SBR. The study by Lv et al. [133] was omitted as no mechanical property data were explicitly reported.

### 5.10. Epoxidised Natural Rubber (ENR)

NR can be chemically modified to make ENR. It is commercially available as 25 mol % epoxidised (ENR25) and 50 mol % epoxidised (ENR50) natural rubber. Epoxidation increases the polarity of the rubber and also increases damping, hysteresis and wet traction. ENR can, unlike NR, be reinforced with silica without using silane coupling agents due to the silanol groups being able to interact with the epoxy groups on ENR and can be cured using both sulphur and peroxide cure systems [47].

Two articles relevant to the search criteria have been published on LDH in ENR, one in 2017 and one in 2020. In both cases Mg-Al LDH was used.

Da Silva et al. [135] added unmodified Mg-Al LDH to ENR and found that the crosslink density and curing behaviour was not affected by the presence of the LDH at any of the loadings they tested (up to 5 phr). The maximum torque increased very slightly at 5 phr LDH. The LDH also decreased the onset temperature of thermal degradation due to the degradation of interlayer carbonates and hydroxyl groups of the LDH.

Li et al. [136] found that by adding dodecyl sulphate-modified Mg-Al LDH (DS-LDH) to ENR, self-healing behaviour can occur. The DS-LDH could also form a hydrogen-bonded network with ENR. DS-LDH was well dispersed and intercalation of ENR chains could occur even at 20 phr DS-LDH, but unmodified LDH formed agglomerates due to incompatibility with the ENR matrix. The hydroxyl groups on the LDH underwent a ring-opening reaction with the oxirane groups in ENR, which allowed covalent crosslinking to take place. The hydroxyl groups also formed hydrogen bonds with the oxygen-containing groups of ENR and suggested the formation of a dynamic hydrogen-bonded supramolecular network. These hydrogen bonds could break and re-form during deformation which increased the residual strain and hysteresis of ENR through increased energy dissipation. The hydrogen bonds also increased the crosslink density. The TS and elastic modulus were increased with increasing DS-LDH content due to the reinforcing effect of the LDH platelets and hydrogen bonding with the rubber matrix that increased energy dissipation as the hydrogen bonds were broken and re-formed. EB was also increased but started to decrease at higher loadings of DS-LDH due to the chain movement becoming too restricted. The self-healing behaviour that was observed was due to the ability of the hydrogen bonds to break and re-form. The healing efficiency increased with increasing DS-LDH content up to 10 phr and then decreased again. The Young’s modulus and EB were increased by 338% and 13%, respectively, at an LDH loading of 20 phr.

### 5.11. Styrene–Ethylene–Butylene–Styrene (SEBS)

SEBS is a thermoplastic elastomer [137]. SEBS forms physical crosslinks. The styrene and ethylene–butylene blocks are incompatible so they phase separate and the ethylene–butylene blocks are locked into a physically crosslinked network structure. SEBS is difficult to chemically crosslink because the polymer chains are hydrogenated, but it is possible to use peroxide cure to crosslink SEBS by first modifying it with maleic anhydride [138].

One article, published in 2018, satisfied the search criteria on the effect of LDH on SEBS and is discussed here.

The interactions between the inorganic LDH and organic elastomer matrix plays an important role in the mechanical properties of the composite. Better interfacial interaction and dispersion, and increased interlayer spacing that enables polymer chain intercalation all help to improve the mechanical properties of the composite [13,75,93,108]. Modifying the polymer by adding polar groups could also increase the interaction between the LDH and polymer. Arrigo et al. [139] investigated the interaction between LDH and the polymer matrix by adding Mg-Al LDH modified with hydrogenated fatty acid to maleic anhydride grafted SEBS (SEBSgMA) and SEBS. They found that there was strong interaction between the LDH and maleic anhydride groups on SEBSgMA. Carboxylic groups and carboxylate salt groups formed via this interaction. The LDH was exfoliated. The interaction between the LDH and anhydride functional groups caused an increase in complex viscosity of the composite. The shear relaxation behaviour of SEBSgMA was affected by the LDH and a crosslinked-like structure was obtained due to the ionic interactions between the LDH and polymer preventing chain relaxation. In SEBS intercalation was favoured more than exfoliation. The complex viscosity was also increased by the LDH. In both SEBS and SEBSgMA, the modified LDH was well dispersed. The combination of organically modified LDH and modified SEBS increased the interactions between the filler and polymer which led to changes in the rheological behaviour of the composites.

### 5.12. Polybutadiene Rubber (PBR)

PBR is a synthetic rubber made from the butadiene monomer in different variations such as high cis content, low or medium cis content, vinyl BR, and emulsion BR [68]. It can be cured using sulphur vulcanisation or peroxide cure [140] and is usually blended with other polymers [68].

The literature review revealed one study conducted on the effect of LDH in PBR, which was published in 2021.

Moshkriz et al. [141] investigated the effect of organically modified LDH on the properties of a thermoplastic vulcanisate, a 60:40 weight ratio PBR/PP blend. PP and PBR are not compatible despite both being non-polar, which reduces the mechanical properties. The Mg-Al LDH was first coated with 3-aminopropyl triethoxysilane (APTES). The modified PBR and PP were then grafted into the silane-modified LDH (Si-LDH). PP and liquid PBR grafted with maleic anhydride were used as compatibilisers in the rubber matrix and grafted onto the LDH (O-LDH). The O-LDH was then added to the PBR/PP blend along with a maleic anhydride compatibiliser, Comp-g-MA (PP and liquid PBR grafted with maleic anhydride), in varying ratios. Increasing the Comp-g-MA content increased the gel content of the polymer blend because the compatibiliser decreases interfacial tension and increases interfacial adhesion between PP and PBR. Incorporating O-LDH and Comp-g-MA increased the crosslink density, EB, TS, Young’s modulus and thermal stability of the polymer blend, though at higher loadings the properties started to decrease again. Both O-LDH and Comp-g-MA had a compatibilising effect in the polymer blend, which enhanced the mechanical properties. The O-LDH had a reinforcing effect in the polymer and increased the storage modulus. The increased crosslink density and interaction between the O-LDH and polymer matrix also caused an increase in T${}_{g}$ due to the polymer chain movement being restricted. The organically modified LDH in combination with the maleic anhydride compatibiliser improved the properties of the PBR/PP blend.

The LDH caused an increase of 156% in TS and 171% in EB at a loading of 5.784 wt % LDH, showing that the mechanical properties of PBR can be improved by adding LDH.

## 6. Conclusions

Since the 2000s LDHs in elastomers have been attracting attention. In general, LDH can be used as a reinforcing filler that enhances the mechanical properties such as TS and EB, although in the case of EB LDH can also cause a decrease due to restricting chain movement and increasing stiffness. LDH platelets tend to have large surface areas and high aspect ratios, providing a large area for filler–polymer interaction and allowing efficient stress transfer. The particles also interrupt crack propagation by acting as physical barriers that cause primary cracks to branch off.

LDH can also affect other properties such as the T${}_{g}$. The T${}_{g}$ can be increased when there is strong interaction between the LDH and polymer matrix, causing chains to become entangled around the LDH particles. This typically occurs when the LDH is organically modified to make it more compatible with the polymer matrix. In polar rubbers an increase in T${}_{g}$ can be observed even with unmodified LDH. Organic modification can, however, have the opposite effect and decrease T${}_{g}$ due to the modifiers having a plasticising effect.

Organic modification of the LDH is usually necessary, especially in nonpolar elastomers, to improve compatibility between the LDH and elastomer. Organic modifiers also increase the interlayer spacing of the LDH, allowing polymer chains to intercalate into the LDH interlayer as well as increasing the exfoliation of the LDH in the polymer matrix. Without organic modification the poor interaction between the LDH and elastomer can reduce the reinforcing effect of the LDH. Intercalation and exfoliation is also more limited. In polar elastomers such as XNBR the LDH can hydrogen bond with the polar groups on the elastomer, which allows good polymer–filler interaction despite the hydrophilic nature of LDH. Organic modification can still be beneficial in polar elastomers and further increase the polymer–filler compatibility and interactions as well as allowing intercalation and exfoliation. The organic modifiers typically used are sodium dodecyl sulphate, sodium dodecyl benzenesulphonate and stearic acid, although others have also been used.

Surface area, aspect ratio and platelet size all have an impact on how the LDH affects the properties of elastomers. In the case of XNBR, there was evidence from two studies that increasing the surface area and Mg content of the LDH increased crosslinking density, cure efficiency and TS. This indicates that the metal ratio and surface area may also affect the properties of elastomers. The type of organic modifier chosen can also have an influence on the properties of the composite. Some modifiers, such as the stearate anion, can cause a plasticising effect and increase the EB and lower T${}_{g}$. Some modifiers seem to be more effective than others. In XNBR, 1-hexadecanesulphonate performed more poorly than 1-decanesulphonate and in NBR styrene sulphonate modification increased the interlayer spacing of the LDH more than dodecyl benzenesulphonate which improved exfoliation and intercalation, for example.

LDH can be combined with other fillers such as CNT and SiO${}_{2}$ as well. Both these fillers agglomerate easily, but it has been shown that, by grafting them onto LDH, dispersion of the hybrid filler is improved and the synergy between the components further improves the properties of the composites. This broadens the possible uses of LDH in elastomers. The LDH and modifier can also be functional in elastomers. Mg-Al LDH has been shown to act as a crosslinker in XNBR that can undergo ionic crosslinking. Zn-Al LDH can be used to replace ZnO in sulphur vulcanisation and in metal oxide crosslinking. Stearate-modified LDH can also be used to replace stearic acid in crosslinking systems. Zn-Al LDH coated in stearic acid has also been used to make a thermotropic SSBR composite and a self-healing ENR composite containing LDH was also synthesised. This again shows that there is a wide range of possible uses for different types of LDH in elastomers. Their easy customisability is part of what enables their versatility.

LDH is typically added to elastomers at concentrations below 10 wt % due to the tendency of LDH to form agglomerates at higher concentrations. Much higher loadings, even up to 100 phr, have also successfully been used. In most cases, the elastomer is reinforced at all concentrations of LDH compared to the neat elastomer but the reinforcement tends to reach a maximum and then decrease again due to the presence of agglomerates. This highlights the importance of ensuring the LDH is uniformly dispersed without agglomerates.

Most of the studies so far have used Mg-Al LDH, with some interest in Zn-Al LDH as well. The only other LDHs that were used were Li-Al LDH and Co-Al LDH, although few studies were done using these two LDHs. The fact that other types of LDH have not been used much leaves scope for further investigations into using different metals and how they affect the mechanical properties of the composites. Elastomers such as NBR, PU, and XNBR have been investigated to a much greater extent than other elastomers such as NR, ENR and CR. While typical trends of reinforcement and effects on properties of LDH seem to be common across most elastomers, it would still be worth investigating the effect of LDH on these elastomers further since elastomers can have different interactions with LDH.

LDHs are clearly a promising reinforcing filler in elastomers and can also act as functional fillers. They are customisable and provide good mechanical properties to the rubber, as long as the LDH can be made compatible with the elastomer, and can at times produce interesting and unique interactions with different types of elastomer. The reinforcing effect may allow LDHs to be used as alternatives to reinforcing fillers such as carbon black and silica that are traditionally used in elastomers. They can also be used as crosslinking agents in the place of traditional crosslinking agents such as MgO and ZnO. This may be useful in applications such as car tire tread, for example, where more environmentally friendly additives and fillers are being investigated. There are not yet many studies that have directly compared the reinforcing effect of LDH to traditional fillers, and as such further exploration of the effect of LDH on the mechanical properties of elastomers may be beneficial.

## Figures and Tables

**Table 1:** Summary of the effect of LDH on the properties of EPDM.

LDH	LDH Loading	% Change					Ref.
		M100	TS	EB	tan(δ) Peak	T${}_{g}$	
Mg${}_{3}$Al-DS	8	+108	+105	+89	-	−4	[52]
Mg${}_{2}$Al-C10	8.5	+10	+111	+127	0	0	[13]
Mg${}_{3}$Al-DS	3	-	+33	+12	−3	+4	[54]
Zn${}_{0.25}$Al-CO${}_{3}$	8.9	+2	+311	+133	−8	+3	[55]
Zn${}_{x}$Al${}_{y}$-St	∼2.5 (3 phr)	-	+37	+18	-	-	[56]

**Table 2:** Summary of the effect of LDH on the properties of XNBR.

LDH	LDH Loading (phr)	% Change					Ref.
		M200	TS	EB	tan(δ) Peak	T${}_{g}$	
Mg${}_{2}$Al${}_{1}$-C10	10	+320	+819	−16	−25	+0	[13]
Mg${}_{2}$Al-CO${}_{3}$	5	-	-	-	−5	-	[57]
Mg${}_{2}$Al-DBS	5	-	-	-	−8	-	
Mg${}_{2}$Al-C10	10	+320	+819	−16	-	-	[59]
Mg${}_{2}$Al-C16	7.5	+232	+586	−12	-	-	
Mg${}_{x}$Al${}_{y}$-A, 5 phr EMIM SCN	30	-	+5	+2	−3	+0	[61]
Mg${}_{x}$Al${}_{y}$-A, 5 phr EMIM TFSI	30	-	+9	+7	+2	−13	
Mg${}_{3}$Al-CO${}_{3}$, 2.5 phr BMIM AlCl${}_{4}$	30	−2	−2	−5	−8	+1	[62]
Mg${}_{3}$Al-CO${}_{3}$, 2.5 phr BMIM TSFI	30	−25	+3	+8	+0	−19	
Mg${}_{3}$Al-CO${}_{3}$, 2.5 phr EMIM TFSI	30	−17	+10	+5	-	−4	[63]
Mg${}_{3}$Al-CO${}_{3}$, 2.5 phr BMIM TFSI	30	−25	+3	+8	-	−4	
Mg${}_{3}$Al-CO${}_{3}$, 5 phr HMIM TFSI	30	−34	−14	+13	-	−13	
Mg${}_{0.6}$Al-CO${}_{3}$	30	+629	+2029	−59	−25	+31	[64]
Mg${}_{2.2}$Al-CO${}_{3}$	30	+600	+2757	−56	−24	+31	
Mg${}_{3}$Al-CO${}_{3}$	30	+1386	+3443	−63	−31	+32	
Mg${}_{3}$Al-CO${}_{3}$	30	+729	+2557	−63	−27	+31	[58]
Zn${}_{x}$Al${}_{y}$-A	20	-	-	-	−9	+22	[17]
Mg${}_{0.07}$Al-CO${}_{3}$	30	-	-	-	+39	−608	[66]

**Table 3:** Summary of the effect of LDH on the properties of CR.

LDH	LDH Loading (phr)	% Change					Ref.
		M300	TS	EB	tan(δ) Peak	T${}_{g}$	
Mg${}_{2}$Al-CO${}_{3}$	5	+51	−4	−25	+20	+2	[69]
Mg${}_{2}$Al-DBS	5	+46	+10	−56	−2	+2	

**Table 4:** Summary of the effect of LDH on the properties of PU.

LDH	LDH Loading (wt %)	% Change						Ref.
		M200	M300	TS	EB	tan(δ) Peak	T${}_{g}$	
Mg${}_{3}$Al-DS	3	-	+15	+67	+27	-	-	[71]
Mg${}_{3}$Al-DS	1	-	+90	+156	+21	-3	+5	[41]
Mg${}_{3}$Al-DS	3	-	-	+407	+176	−26	+54	[72]
Mg${}_{3}$Al-St	1	-	+10	+45	+36	−8	+4	[73]
MDI-Mg${}_{3}$Al-NO${}_{3}$	3	-	+7	+125	+28	-	+1	[75]
IPDI-Mg${}_{3}$Al-NO${}_{3}$	3	-	+4	+60	+36	-	+0	
Mg${}_{3}$Al-DS	3	-	+19	+191	+38	-	+7	
Mg${}_{3}$Al-St	3	-	+24	+136	+43	-	+5	
MDI-Mg${}_{3}$Al-DS	3	-	+48	+270	+39	-	+14	
MDI-Mg${}_{3}$Al-St	3	-	+111	+391	+31	-	+26	
IPDI-Mg${}_{3}$Al-DS	3	-	+30	+241	+44	-	+10	
IPDI-Mg${}_{3}$Al-St	3	-	+56	+318	+45	-	+19	
Co${}_{2}$Al-DS	2	-	-	+150	−7	-	-	[76]
Mg${}_{3}$Al-St	3	-	+75	+222	+14	−10	+15	[77]
D-Mg${}_{3}$Al-CO${}_{3}$, small	4	-	-	-	+3	-	+7	[79]
D-Mg${}_{3}$Al-CO${}_{3}$, large	4	-	-	-	+4	-	+5	
Mg${}_{3}$Al-DS	1	-	-	-	-	−11	+25	[80]
Mg${}_{3}$Al-CO${}_{3}$, T	-	-	-	-	-	+50	+9	[81]
Mg${}_{3}$Al-CO${}_{3}$, TBT	-	-	-	-	-	82	+7	
APS-Mg${}_{2}$Al-DS	3	-	-51	+24	+430	+9	−9	[82]
CNT-DS-Mg${}_{3}$Al-CO${}_{3}$	0.5	+33	-	+171	+75	−22	+6	[85]
CNT-DS-Zn${}_{3}$Al-NO${}_{3}$	0.5	+35	-	+126	+50	−16	+17	[86]
CNF-DS-Zn${}_{3}$Al-NO${}_{3}$	0.5	+44	-	+122	+43	−10	+11	
CNF-DS-Mg${}_{3}$Al-NO${}_{3}$	0.5	-	+64	+167	+52	−5	+8	[87]
Mg${}_{2}$Al-NO${}_{3}$	4	+4	-	−57	−81	-	-	[88]
Mg${}_{2}$Al-DS	5	-	-	+60	+19	-	−25	[89]

**Table 5:** Summary of the effect of LDH on the properties of NR.

LDH	LDH Loading (phr)	% Change					Ref.
		M300	TS	EB	tan(δ) Peak	T${}_{g}$	
Zn${}_{3}$Al-DS	7	-	+28	-	-	-	[93]
Mg${}_{x}$Al${}_{y}$-A	3	+7	−41	−21	-	-	[94]
Mg${}_{x}$Al${}_{y}$-HFA	3	+11	−20	−16	-	-	
Mg${}_{3}$Al-CO${}_{3}$	30	+40	+16	−25	−12	+1	[95]
Mg${}_{3}$Al-CO${}_{3}$, 3 phr BmiBr	30	+144	+37	−37	−16	−3	
Mg${}_{3}$Al-CO${}_{3}$, 3 phr BmpyrBr	30	+100	+25	−28	−20	−4	

**Table 6:** Summary of the effect of LDH on the properties of SR.

LDH	LDH Loading (wt %)	% Change					Ref.
		M100	TS	EB	tan(δ) Peak	T${}_{g}$	
Mg${}_{3}$Al-DS	5	-	+53	+39	-	-	[98]
Mg${}_{3}$Al-St	3	+51	+103	+46	−40	−1	[99]
MWCNT-Mg${}_{3}$Al-NO${}_{3}$	1	90	+134	+52	-	+4	[83]
MWCNT-Li${}_{3}$Al-NO${}_{3}$	1	93	+113	−1	-	+2	
MWCNT-Co${}_{3}$Al-NO${}_{3}$	1	90	+125	+8	-	+2	
Mg${}_{2.1}$Al-CO${}_{3}$, 2.1 wt % ViTES	3.2	-	−8	−7	-	-	[100]

**Table 7:** Summary of the effect of LDH on the properties of NBR.

LDH	LDH Loading (phr)	% Change						Ref.
		M200	M300	TS	EB	tan(δ) Peak	T${}_{g}$	
Mg${}_{3}$Al-DS	1.1	+67	-	+156	+21	−3	+5	[41]
Mg${}_{2-x}$Zn${}_{x}$Al-St	4	+1	-	−1	+2	-	-	
Mg${}_{2-x}$Zn${}_{x}$Al-St, carbon black	4	+4	-	+10	−4	-	-	[16]
Mg${}_{3}$Al-NO${}_{3}$	3	-	-	−90	−8	-	+2	[108]
Mg${}_{3}$Al-SS	5	-	-	+116	−6	-	+5	
Mg${}_{3}$Al-DBS	5	-	-	+16	−4	-	+5	
Mg${}_{2}$Al-NO${}_{3}$	30	-	+38	+241	+5	-	−9	[111]
Mg${}_{2}$Al-LS	30	-	+376	+428	+6	-	−4	
Zn${}_{x}$Al${}_{y}$-St, sulphur cure, low ACN	5	+44	-	+17	−16	-	-	[112]
Zn${}_{x}$Al${}_{y}$-St, sulphur cure, high ACN	5	+12	-	−49	−7	-	-	
Zn${}_{x}$Al${}_{y}$-St, peroxide cure, low ACN	5	+36	-	+14	−7	-	-	
Zn${}_{x}$Al${}_{y}$-St, peroxide cure, high ACN	5	+31	-	+41	−8	-	-	
Mg${}_{3}$ZnAl${}_{2}$-CO${}_{3}$	5	+45	+85	+176	+20	-	-	[113]
Zn${}_{2}$Al-St, commercial	5	+18	+31	+41	+13	-	-	
Zn${}_{2}$Al-St, lab	5	+18	+23	+53	+9	-	-	
CNT-DS-Mg${}_{3}$Al-NO${}_{3}$	0.51	+33	-	+171	+75	−22	+6	[85]
CNT-DS-Zn${}_{3}$Al-NO${}_{3}$	0.51	+35	-	+126	+50	−16	+17	[86]
CNF-DS-Zn${}_{3}$Al-NO${}_{3}$	0.51	+44	-	+122	+43	−10	+11	
CNF-DS-Mg${}_{3}$Al-NO${}_{3}$	0.51	+64	-	+167	+52	−5	+8	[87]
Mg${}_{3}$Al-NO${}_{3}$	1	-	-	+11	+7	-	-	[114]
Mg${}_{3}$Al-SSS	1	-	-	+12	+0	-	-	
PVP-Mg${}_{2}$Al-NO${}_{3}$	-	-	-	+14	−16	-	-	[115]
Mg${}_{3}$Al-NO${}_{3}$	5	-	+12	+15	+17	−5	+16	[116]
Mg${}_{3}$Al-SAS	5	-	+8	+10	+9	−8	+10	
Mg${}_{3}$Al-CO${}_{3}$, BMpyrrolBF4	30	-	-	-	-	−10	+10	[117]
Mg${}_{3}$Al-CO${}_{3}$, BMpyrBF4	30	-	-	-	-	−9	+10	
Mg${}_{3}$Al-CO${}_{3}$, BMpipBF4	30	-	-	-	-	−15	+10	
KH560-RT-GO-Mg${}_{3}$Al-NO${}_{3}$	7	-	+16	+103	+16	+1	+0	[118]

**Table 8:** Summary of the effect of LDH on the properties of HNBR.

LDH	LDH Loading (phr)	% Change						Ref.
		M200	M300	TS	EB	tan(δ) Peak	T${}_{g}$	
IA-Mg${}_{3}$Al-CO${}_{3}$	9	-	+11	+59	−1	+0	+10	[124]
SiO${}_{2}$-Zn${}_{2}$Al-CO${}_{3}$	0.92	+109	-	+1164	+86	+40	+10	[127]

**Table 9:** Summary of the effect of LDH on the properties of SBR and SSBR.

LDH	LDH Loading (phr)	% Change						Ref.
		M100	M300	TS	EB	tan(δ) Peak	T${}_{g}$	
Zn${}_{3}$Al-St	80	+12	-	+131	+49	−48	+0	[132]
Lignin-Mg${}_{2}$Al-NO${}_{3}$ (15 phr lignin)	30	-	+41	+445	+115	-	+10	[110]
SiO${}_{2}$-DBS-Mg${}_{2}$Al-CO${}_{3}$	80	+507	-	+1411	+104	-	-	[125]
SiO${}_{2}$-Mg${}_{2}$Al-CO${}_{3}$	11.7	+113	-	+1551	+104	+29	+0	[126]
